# Naphthalene‐Modified Cationic Initiators for Superior Polymerization Stability and Thermal Conductivity in Epoxy Thermosets

**DOI:** 10.1002/smsc.202500625

**Published:** 2026-03-06

**Authors:** Yewon Woo, Yeonha Ju, Naye Hong, Kyeong Pang, Mooho Lee, In Kim, Munju Goh

**Affiliations:** ^1^ Department of Chemical Engineering Konkuk University Seoul Republic of Korea; ^2^ Material Research Center Samsung Advanced Institute of Technology Samsung Electronics Co., Ltd Suwon Republic of Korea

**Keywords:** cationic initiators, imine moiety, liquid crystalline epoxy, naphthalene‐modified cations, thermal conductivity

## Abstract

Naphthalene‐modified cationic initiators with tunable counteranions were developed to extend the epoxy curing lifetime, enabling the efficient formation of high‐ordered networks in both conventional and liquid crystalline (LC) epoxy systems. The extended lifetime of these initiators allows the LC epoxies to maintain their inherently ordered structure during network formation, which is essential for efficient phonon transport. Differential scanning calorimetry demonstrated a broader exothermic peak width and a more uniform heat release profile, confirming controlled and efficient polymerization. X‐ray diffraction analysis further demonstrated that the π–π stacking peak characteristic of the LC domains was clearly preserved, indicating that the mesogenic order was maintained after curing. Consequently, the cured LC epoxies exhibited a remarkably high thermal conductivity of 0.86 W m^−1^ K^−1^. This is one of the highest reported for unfilled organic systems. Adding hexagonal boron nitride (*h*‐BN) filler further enhanced heat transfer, reaching 25.08 W m^−1^ K^−1^ at 85 wt%. These results demonstrate that rational initiator design with long‐term operation and controlled polymerization provides a simple strategy for fabricating high‐purity, thermally conductive, and reprocessable epoxy networks for advanced thermal management applications.

## Graphical abstract

Naphthalene‐modified cationic initiators extend epoxy curing time, preserving intrinsic LC ordering and achieving exceptionally high thermal conductivity of 0.86 W m^−1^ K^−1^ in unfilled networks. Incorporating thermally conductive fillers further boosts performance, yielding composite conductivities up to ~25 W m^−1^ K^−1^.

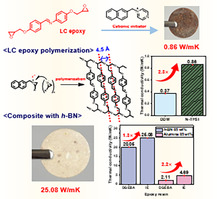

## 1 Introduction

Thermal management materials play a critical role inensuring the reliability and performance of modern electronic devices, which require efficient heat dissipation as power density and integration increase [1]. Among these materials, epoxy systems, particularly those used in epoxy molding compounds, are widely used due to their mechanical strength, dimensional stability, and processability [2]. However, their inherently low thermal conductivity significantly limits their heat dissipation effectiveness, even when high amounts of thermally conductive inorganic fillers are added [3]. This limitation arises because the overall thermal performance of composites is fundamentally limited by the polymer matrix itself, regardless of filler content [4, 5]. Because phonon transport is highly dependent on the molecular structure, bonding, and alignment within the resin, epoxy systems with intrinsic thermal conductivity are increasingly required for advanced electronic packaging.

To address these challenges, cationic polymerization has been explored as an effective strategy for generating highly ordered epoxy networks that facilitate phonon transport. During the curing process, cationic initiators promote rapid, controlled ring‐opening polymerization, allowing mesogenic monomers or segments to self‐organize and align before the network solidifies [6, 7]. This process creates anisotropic structures that provide efficient thermal conduction pathways by forming ordered domains through molecular interactions such as *π–* *π* stacking and dipole alignment [8]. Consequently, the thermal conductivity of epoxy thermosets prepared with conventional initiators is typically only 0.48 W m^−1^ K^−1^, even in liquid crystalline (LC) systems, suggesting that the potential of mesogenic alignment for heat transfer has not yet been fully realized [8]. This demonstrates the urgent need for novel initiator systems that can achieve higher thermal conductivities through enhanced structural alignment.

Designing cationic initiators with enhanced stability and precise molecular structures is crucial for achieving stable and homogeneous polymerization. Introducing a rigid polycyclic aromatic backbone into the cationic salt structure enhances crystallinity and thermal stability, thereby enhancing initiator performance during the curing process [9]. Furthermore, the choice of counter anion plays a critical role in controlling the strength of ionic interactions and the overall reactivity of cationic initiator systems [10, 11]. These molecular design factors allow for precise control of the polymerization kinetics and the formation of more homogeneous and ordered epoxy networks. To further enhance thermal conductivity, LC epoxy monomers offer a unique advantage by promoting directional phonon transport through self‐assembled molecular alignment [12, 13, 14]. However, this alignment is highly dependent on the kinetics of the curing process, which are determined by the properties of the initiator. By incorporating well‐designed cationic initiators with LC monomers, anisotropic network structures with superior thermal performance can be constructed.

In this study, we report the rational design of naphthalene‐modified cationic initiators with tunable counter anions to enhance both the efficiency and lifetime of epoxy curing. These initiators provide extended operating times, allowing the LC epoxy to maintain its inherently ordered structure during network formation. As a result, the cured liquid crystal epoxy achieves a very high thermal conductivity of 0.86 W m^−1^ K^−1^, which is exceptional for an unfilled organic material. The extended initiator lifetime ensures more uniform and efficient curing, while maintaining the inherent order of the liquid crystal domains, which is essential for efficient phonon transport. Combining controlled polymerization and long‐operating times, this naphthalene‐modified initiator provides a simple yet powerful strategy for achieving highly ordered and thermally conductive epoxy networks, which have previously been difficult to achieve in purely organic systems.

## 2 Results and Discussion

### 2.1 Synthesis of the Naphthalene‐Modified Cationic Initiator and Curing Mechanism

**SCHEME 1:**
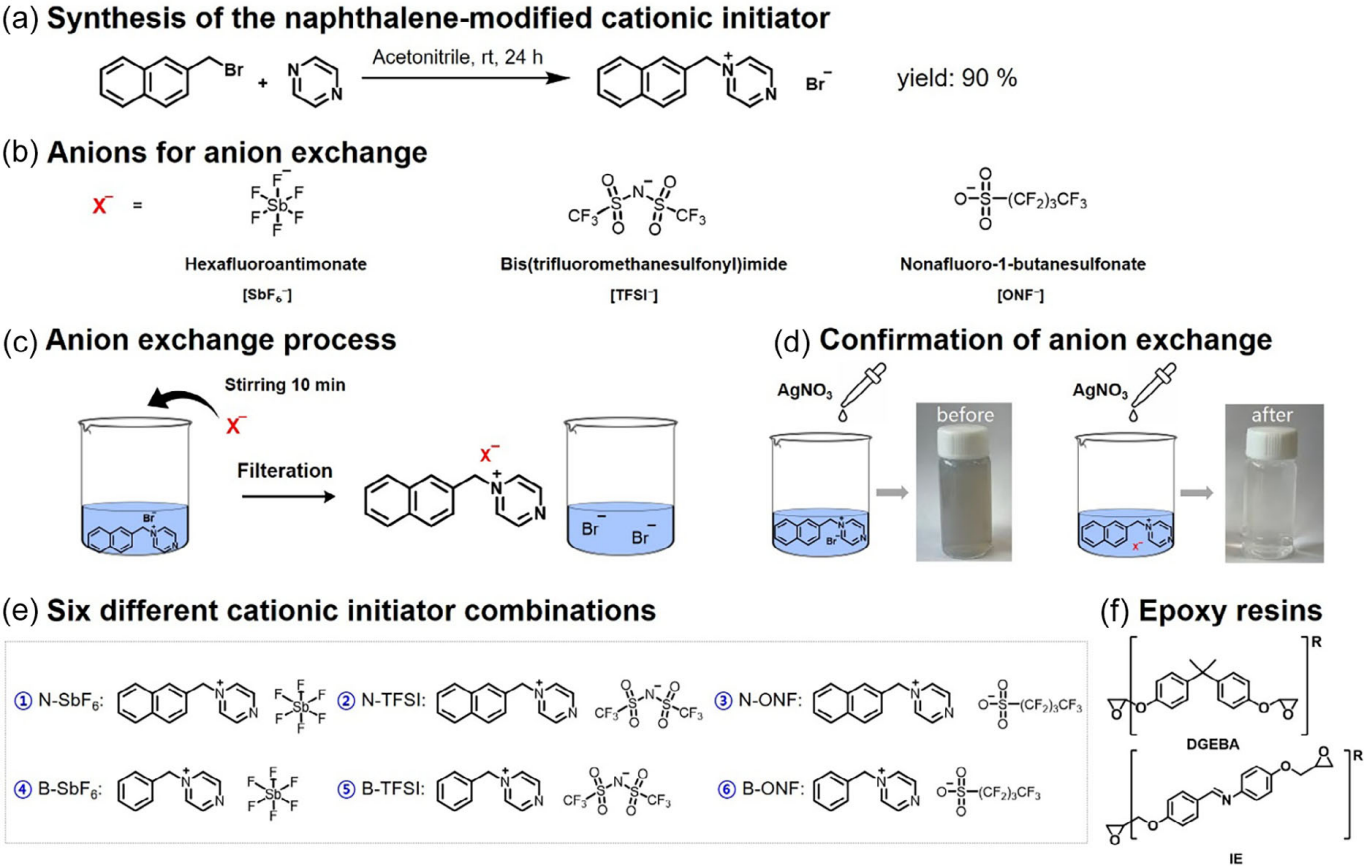
(a) Synthetic route of the naphthalene‐modified cationic initiator; (b) Counteranions used: hexafluoroantimonate (SbF_6_^−^), bis(trifluoromethanesulfonyl)imide (TFSI^−^), and nonafluoro‐1‐butanesulfonate (ONF^−^); (c) Anion‐exchange process; (d) Confirmation of anion exchange using AgNO_3_; (e) Six different cationic‐initiator combinations obtained; (f) Chemical structures of the epoxy resins used in this study: diglycidyl ether of bisphenol A (DGEBA) and imine‐functionalized liquid crystalline epoxy (IE).

To develop a thermally latent cationic initiator, a naphthalene‐modified pyrazinium salt was synthesized via nucleophilic substitution between 2‐(bromomethyl)naphthalene and pyrazine (Scheme 1a), and its formation was confirmed by ^1^H NMR spectroscopy (Figure S1a). Similarly, benzyl bromide and pyrazine were used to synthesize benzyl‐based pyrazinium cationic initiators and its formation was confirmed by ^1^H NMR spectroscopy (Figure S1b).

The halide anion of the as‐synthesized salt was replaced with three nonnucleophilic counteranions—hexafluoroantimonate (SbF_6_^−^), bis(trifluoromethanesulfonyl)imide (TFSI^−^), and nonafluoro‐1‐butanesulfonate (ONF^−^)—to investigate how distinct anionic motifs—antimony‐centered, oxygen‐based sulfonate, and nitrogen‐centered imide—affect the electronic environment, thermal stability, and reactivity of the cationic initiator [7]. Bromonaphthalene pyrazinium was dissolved in distilled water, and each anion was added in a 1:1.2 molar ratio (pyrazinium:anion). The mixtures were stirred at room temperature for 10 min, and the products were isolated by filtration or precipitation, washed, and dried under vacuum. Completion of ion exchange was confirmed by silver nitrate precipitation tests, in which the absence of a visible precipitate indicated full removal of halide ions [7]. (Scheme 1d). Using these counteranions, six different cationic‐initiator combinations were obtained (Scheme 1e).

Finally, diglycidyl ether of bisphenol A (DGEBA) and an imine‐functionalized LE epoxy (IE) were employed as model epoxy resins to evaluate the influence of the stabilized cationic initiators on curing behavior and network formation (Scheme 1f). The IE resin was synthesized via condensation of 4‐hydroxybenzaldehyde and 4‐aminophenyl, followed by epoxidation using epichlorohydrin, according to previously reported procedures [14]. Successful synthesis was confirmed by ^1^H NMR spectroscopy (Figure S1c), showing distinct proton signals for the imine linkage and terminal epoxide groups.

**SCHEME 2:**
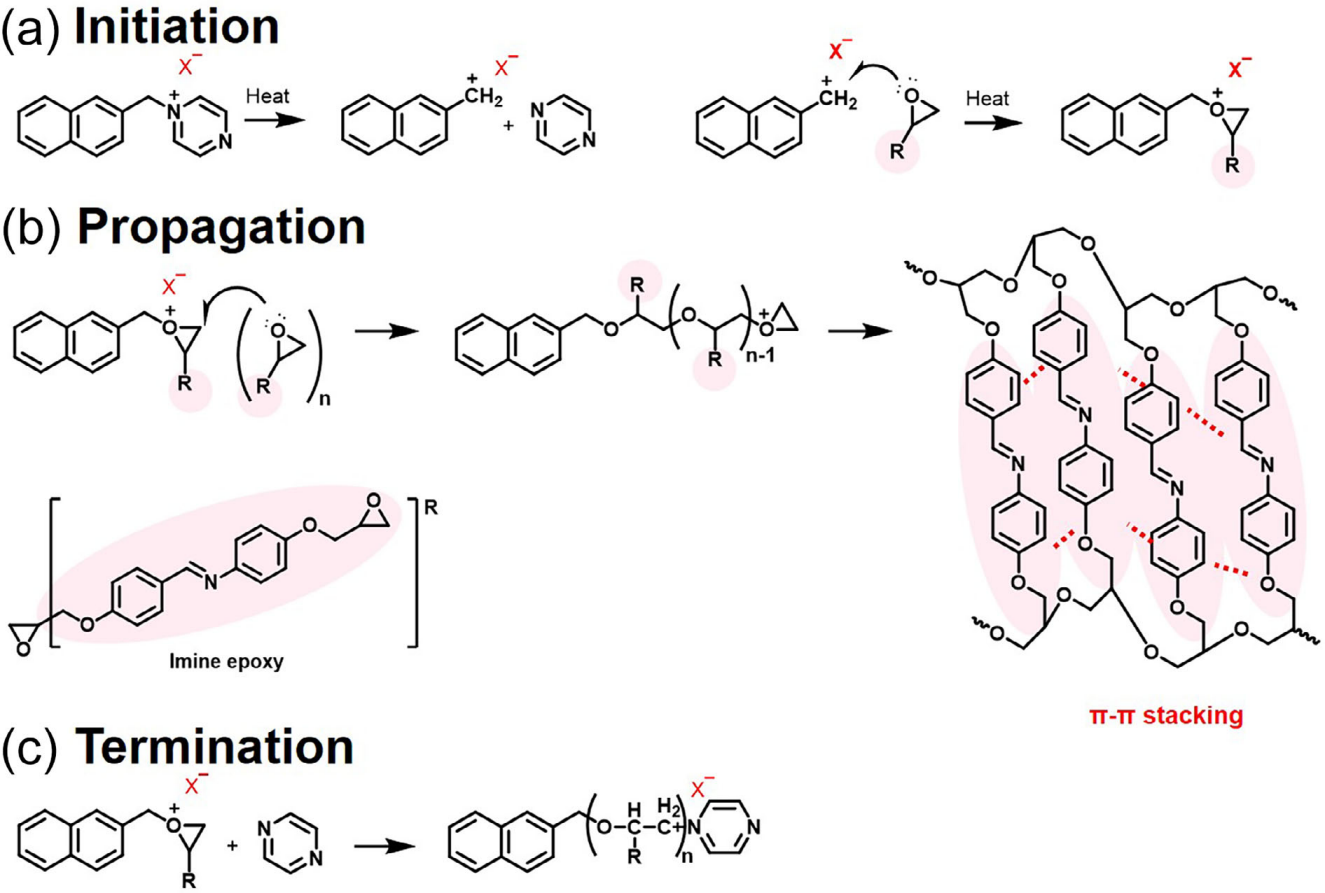
Curing reaction pathway of epoxy resin initiated by a naphthalene‐modified cationic initiator: (a) Thermal activation of the cationic initiator generating active cationic species, (b) ring‐opening polymerization of epoxy groups leading to chain propagation and network formation, and (c) termination of the polymerization.

Curing of the epoxy resin using the synthesized cationic initiator proceeded through a thermally latent cationic ring‐opening polymerization mechanism (Scheme 2). Upon thermal activation, thermal decomposition of the cationic initiator generated a carbocation modified with naphthalene, which acted as an electrophilic center and attacked the electron‐rich epoxide ring. This initiation reaction triggered a series of ring‐opening reactions that propagated along the polymer chain. Polymerization not only progressed primarily through linear chain growth but also involved extensive interchain crosslinking, forming a three‐dimensional crosslinked polymer network.

Importantly, the addition of a bulky, conjugated naphthalene moiety not only enhanced the initiator's lifetime and kinetic stability but also improved molecular alignment during curing. Specifically, in the presence of the liquid‐crystal epoxy monomer, the ordered arrangement of the mesogenic units was maintained or even promoted during polymerization. This enhanced molecular alignment minimized phonon scattering within the polymer matrix, facilitating more efficient heat transfer. Consequently, the final cured network exhibited significantly improved thermal conductivity compared to networks cured with conventional initiators. The cation propagation is terminated through reaction with a neutral nucleophile such as pyrazine, stabilizing the system and completing network formation.

### 2.2 Effect of Initiator Structure and Counteranions on Crosslinking and Thermal Conductivity

**FIGURE 1:**
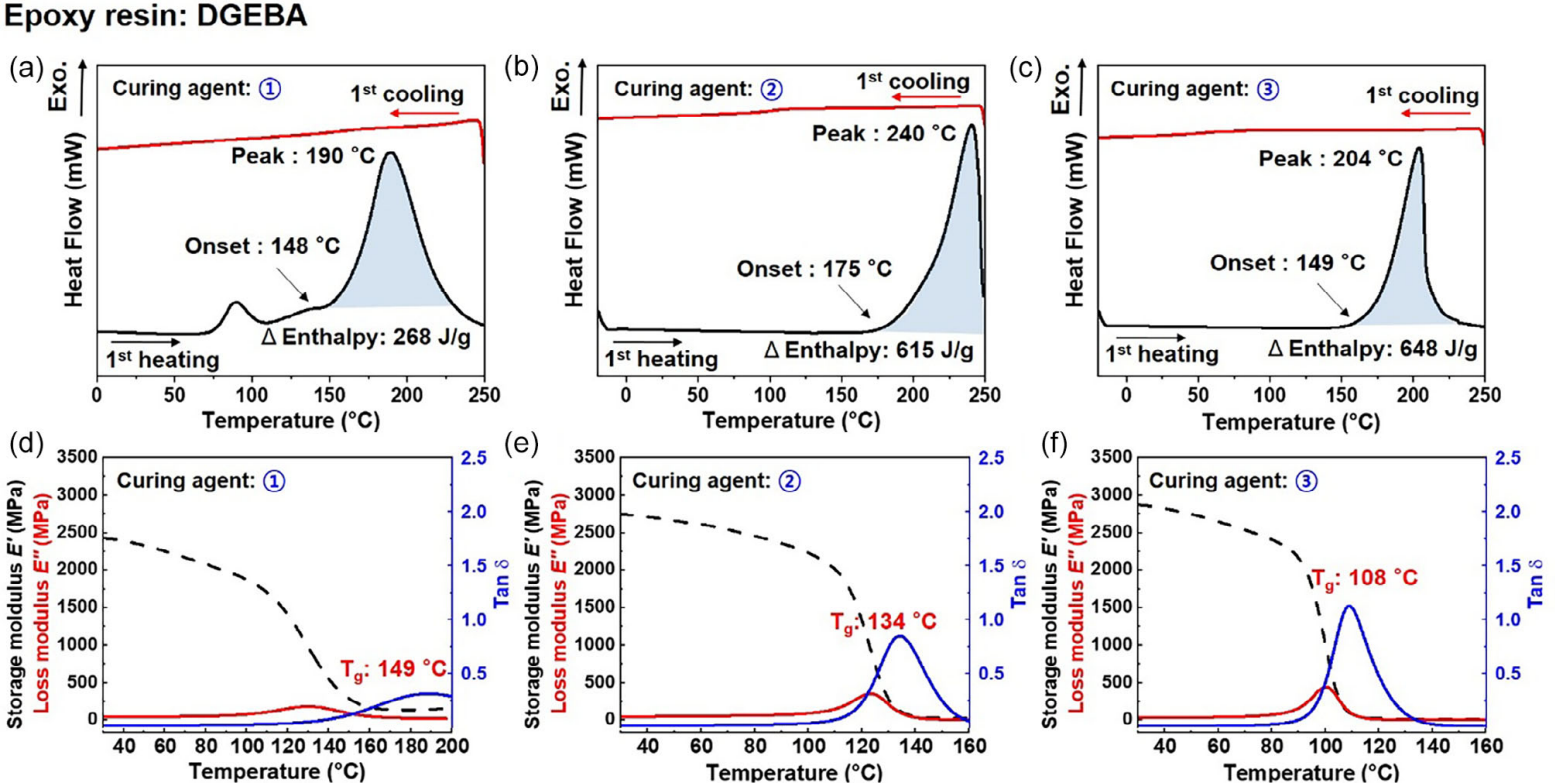
(a–c) DSC first heating and cooling curves of DGEBA epoxy systems containing each naphthalene‐modified cationic initiator, measured under a nitrogen atmosphere at a heating/cooling rate of 10°C·min^−1^ (a) Naphthalene‐modified hexafluoroantimonate (N‐SbF_6_), (b) Naphthalene‐modified bis(trifluoromethanesulfonyl)imide (N‐TFSI), (c) Naphthalene‐modified nonafluoro‐1‐butanesulfonate (N‐ONF). (d–f) Storage and loss modulus as a function of time for DGEBA epoxy systems with each initiator, measured while increasing the temperature at a rate of 3°C ·min^−1^ (d) N‐SbF_6_, (e) N‐TFSI, and (f) N‐ONF.

To determine the appropriate curing condition for DGEBA epoxy systems using naphthalene‐modified cationic initiators, differential scanning calorimetry (DSC) analysis was conducted. The N‐SbF_6_ exhibited an onset curing temperature of approximately 148°C, with a peak exotherm at 190°C and an enthalpy of 268 J/g (Figure 1a). Interestingly, the DSC thermogram of N‐SbF_6_ showed an additional small peak near 70°C, which is attributed to the thermal activation of the cationic initiator. This observation suggests that the initiator activation temperature is distinct from the actual curing temperature of the epoxy system. Similar shoulder features were also observed in the DSC curves of N‐TFSI, and N‐ONF, indicating overlapping but separate stages of initiator activation and epoxy crosslinking. Among the initiators, N‐TFSI exhibited the highest onset and peak temperatures at 175°C and 240°C, respectively, with an enthalpy of 615 J/g (Figure 1b). N‐ONF initiated curing at 149°C, reached a peak temperature of 204°C, and exhibited the highest enthalpy of the three, 648 J/g (Figure 1c). Although the curing behavior varied, for comparison, all samples were cured isothermally at 180°C, between the initiation and peak temperatures of the initiators. This DSC analysis demonstrates that the naphthalene‐modified cationic initiator exhibited a significantly higher polymerization enthalpy than the benzyl‐based initiator, suggesting a more exothermic and energetically favorable curing behavior (Figure S2). The enthalpy increase is attributed to the *π*‐conjugated expansion of the naphthalene moiety, which stabilizes the active cationic species during the propagation process and promotes the formation of a denser and more homogeneous cross‐linked network.

Subsequent dynamic mechanical analysis (DMA) was performed to evaluate the viscoelastic properties of the cured epoxy networks. Among the systems, N‐SbF_6_ exhibited the highest glass transition temperature (*T*_g_) at 149°C, followed by N‐TFSI (134°C) and N‐ONF (108°C). This trend is closely related to the strength of ionic interactions and the rigidity of the resulting network. N‐SbF_6_ likely forms the most thermally stable network due to the strong ion pairing between the naphthalene‐modified cation and the SbF_6_^−^ anion. The relatively small ionic radius of SbF_6_^−^ increases electrostatic interactions, reducing segment mobility and increasing the *T*_g_. The ion pairing strength decreases in the order of anion size: SbF_6_^−^ < TFSI^−^ < ONF^−^, resulting in a progressively lower *T*_g_ value for the corresponding systems.

The observed differences highlight the influence of anion type on the thermal and mechanical performance of epoxy thermosets.

**FIGURE 2:**
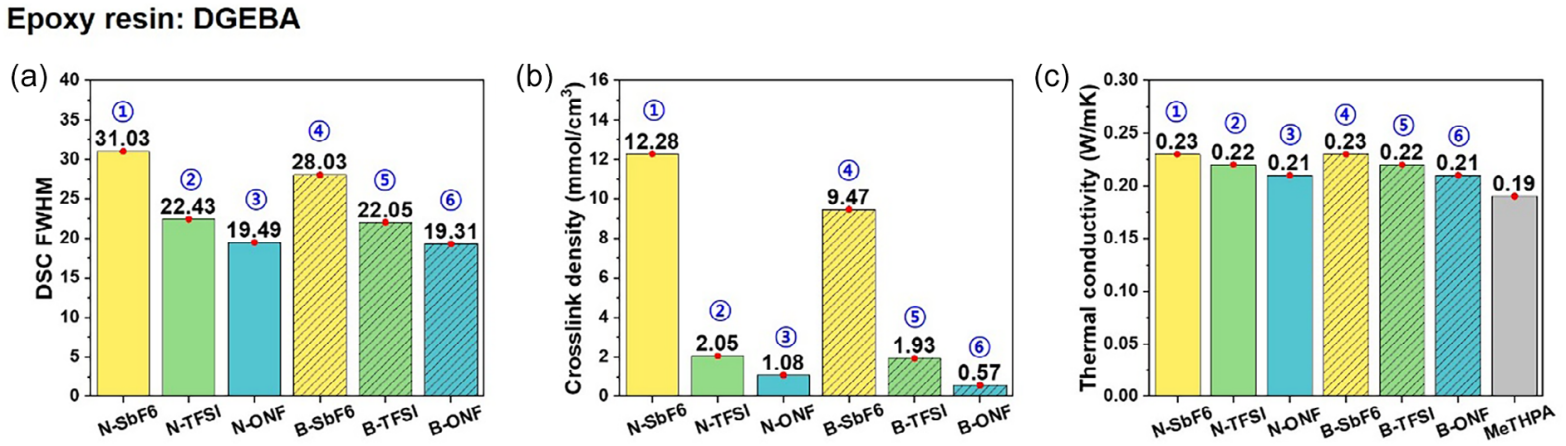
Comparative analysis of the properties of DGEBA cured with each naphthalene‐modified and benzyl‐based cationic initiator: (a) DSC full width at half maximum (FWHM), (b) crosslink density, and (c) In‐plane thermal conductivity, measured using a Hot Disk TPS 2500 (ThermTest Inc., Sweden) in anisotropic mode based on the transient plane source (TPS) method.

To elucidate the influence of counteranion species and aromatic substituents on the thermal transport properties of cured epoxy networks, we systematically compared the DSC full width at half maximum (FWHM) and crosslink densities of samples cured with naphthalene‐ and benzyl‐based cationic initiators bearing three different counteranions (SbF_6_^−^, TFSI^−^, and ONF^−^) (Figure 2).

The DSC thermograms in Figure 1a–c revealed notable variations in FWHM depending on the counteranion type for the naphthalene‐modified initiators. FWHM is defined as the temperature difference between the left and right intersection points at half the peak height. Broader FWHM values indicate prolonged lifetimes of the active cationic species, which in turn extend polymerization initiation activity. Quantitatively, the FWHM values were 31.03, 22.43, and 19.49 for N–SbF_6_, N–TFSI, and N–ONF, respectively, demonstrating a clear hierarchy in initiation persistence (Figure 2a).

Benzyl‐based initiators also exhibited similar trends, with FWHM values of 28.03, 22.05, and 19.43 for B–SbF_6_, B–TFSI, and B–ONF, respectively. The slightly broader FWHM of the naphthalene‐modified initiator indicates enhanced thermal stability and slower deactivation of the active cationic species, which can be attributed to the extended *π*‐conjugation and resonance stabilization provided by the naphthalene substituent. This positive charge delocalization suppresses premature termination and promotes more controlled polymerization (Figure 2a).

This extended initiation activity is directly correlated with a greater number of active initiation sites, which promotes the formation of a denser crosslinking network. The crosslink densities in the rubbery plateau region were calculated by dividing the DMA storage modulus by 3RT (*R*: gas constant, *T*: absolute temperature). The value were 12.28, 2.05, and 1.08 mmol·cm^−3^ for N–SbF_6_, N–TFSI, and N–ONF, respectively, while the benzyl‐based initiators exhibited 9.47, 1.93, and 0.57 mmol·cm^−3^ for the same counteranions (Figure 2b). While the counteranion‐dependent trend was consistent across both aromatic substituents, the naphthalene‐modified initiator formed a noticeably denser network. This dense crosslinking suggests a more stable cationic activity during the curing process, which increases mechanical stiffness and allows for more efficient phonon transport through the charged structure.

To further evaluate the macroscopic impact of these microstructural differences, thermal conductivity measurements were performed using both DGEBA and imine‐functionalized LC epoxy matrices. Although absolute thermal conductivity values varied across epoxy systems, the relative effects of counteranions and aromatic substituents were consistent across both. In DGEBA, naphthalene‐ and benzyl‐based initiators exhibited similar thermal conductivities (Figure 2c), but both exhibited ≈20% higher thermal conductivities than samples cured with the anhydride curing agent, methyltetrahydrophthalic anhydride (MeTHPA) (Figure S3a). This demonstrates the advantage of cationic polymerization in providing a homogeneous and efficient curing pathway, which reduces phonon scattering through more homogeneous crosslinking and denser network formation.

For a more sensitive comparison between naphthalene‐ and benzyl‐based initiators, LC epoxy was used as a platform because the ordered mesophase facilitates directional phonon transport and better demonstrates differences in initiator performance. Overall, these results demonstrate that both the counteranion species and aromatic substituents significantly influence the lifetime and activity of the cationic initiator, thereby modulating the crosslink density, network uniformity, and molecular mobility of the cured epoxy system. This molecular‐level control directly enhances thermal conductivity, highlighting the importance of rationally designing initiator‐counteranion pairs for the development of high‐performance thermal management materials.

### 2.3 Influence of Mesophase Domain Preservation on Thermal Conductivity

**FIGURE 3:**
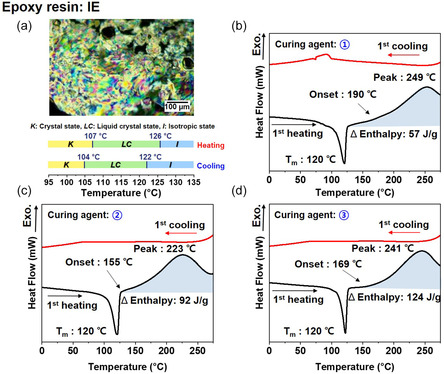
(a) Polarized optical microscopy (POM) image of the imine‐functionalized liquid crystalline epoxy (IE) and its corresponding liquid crystalline temperature range; (b–d) DSC first heating and cooling curves of IE systems containing each naphthalene‐modified cationic initiator, measured under a nitrogen atmosphere at a heating rate of 10°C·min^−1^: (b) N‐SbF_6_, (c) N‐TFSI, and (d) N‐ONF.

To further investigate how the mesophase state influences thermal transport and curing behavior, the imine‐functionalized LE epoxy (IE) was cured using three distinct naphthalene‐modified cationic initiators. The LE nature of IE was confirmed by polarized optical microscopy (POM), which revealed its corresponding LC temperature range between 110°C and 125°C (Figure 3a).

DSC analysis revealed an endothermic peak around 120°C, corresponding to the melting point of IE. The onset and peak temperatures, as well as the associated enthalpy changes, varied depending on the initiator: N‐SbF_6_ (onset temperature 190°C, peak temperature 249°C, Δ*H* 57 J g^−1^), N‐TFSI (onset temperature 155°C, peak temperature 223°C, Δ*H* 92 J g^−1^), and N‐ONF (onset temperature 169°C, peak temperature 241°C, Δ*H* 124 J g^−1^) (Figure 3b–d).

Notably, all three initiators exhibited exothermic curing peaks outside the liquid crystal temperature range, indicating that conventional one‐step thermal curing would inevitably disrupt the mesophase structure during network formation. To overcome this limitation, a two‐step curing protocol was deliberately designed. The pre‐curing temperature of 120°C was chosen to fall within the LC mesophase window (110°C–125°C), where molecular self‐assembly and domain growth can proceed without disturbing the LC order. The post‐curing temperature of 190°C was selected based on the main curing exotherms observed in DSC (Figure 3b–d), in order to ensure complete conversion of residual epoxy groups and full network formation.

Furthermore, the pre‐curing durations of 2 and 24 h were systematically compared to investigate the effect of mesophase preservation time on molecular alignment, network homogeneity, and thermal transport properties. This two‐step curing strategy enables partial network fixation within the LC phase followed by complete crosslinking at elevated temperature, thereby allowing controlled preservation of the mesophase domain structure, which plays a critical role in determining the final thermal conductivity of the epoxy network.

**FIGURE 4:**
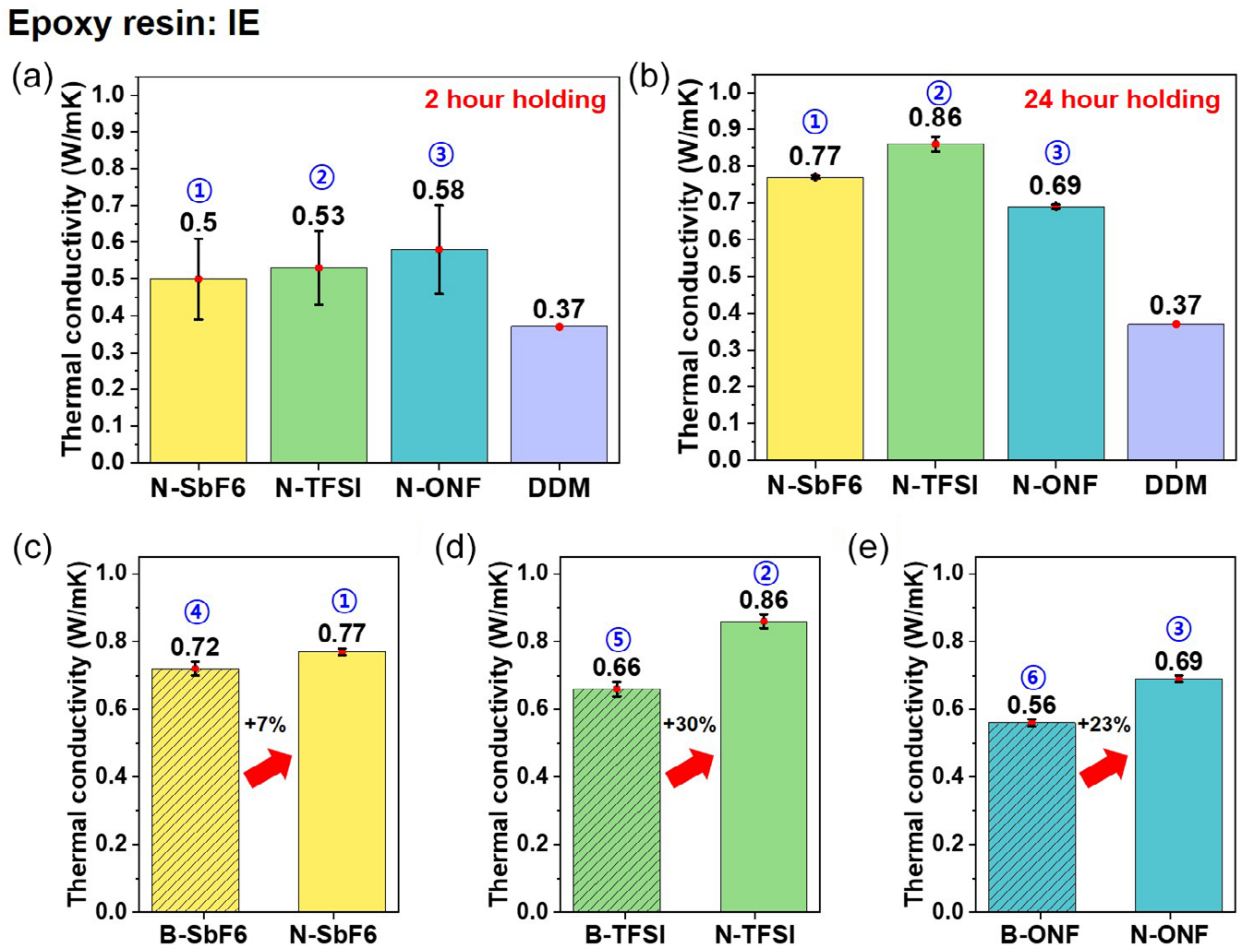
(a,b) Comparative analysis of the in‐plane thermal conductivity of imine‐functionalized epoxy (IE) cured with each naphthalene‐modified cationic initiator and a conventional curing agent, 4,4′‐diaminodiphenylmethane (DDM): (a) after 2 h holding at 120°C (LC state), and (b) after 24 h holding at 120°C. (c–e) In‐plane Thermal conductivity comparison of IE composites cured with naphthalene‐modified and benzyl‐based cationic initiators according to counteranion type: (c) hexafluoroantimonate (SbF_6_^−^), (d) bis(trifluoromethanesulfonyl)imide (TFSI^−^), and (e) nonafluoro‐1‐butanesulfonate (ONF^−^) All thermal conductivity values are presented as mean ± standard deviation obtained from four independently prepared samples (*n* = 4).

Thermal conductivity measurements conducted at a power of 80 mW for 10 s revealed a pronounced dependence on mesophase domain preservation in IE samples cured with naphthalene‐modified cationic initiators. IE samples pre‐cured at 120°C for 2 h exhibited thermal conductivities of 0.50 ± 0.11, 0.53 ± 0.10, and 0.58 ± 0.12 W m^−1^ K^−1^ for N‐SbF_6_, N‐TFSI, and N‐ONF, respectively (*n* = 4), representing up to a 57% increase compared to the IE sample cured with the conventional amine‐based hardener DDM, which had a thermal conductivity of 0.37 W m^−1^ K^−1^, as shown in Figure 4a. Extending the pre‐curing time within the LC phase to 24 hr further enhanced thermal conductivities to 0.77 ± 0.01, 0.86 ± 0.02, and 0.69 ± 0.01 W m^−1^ K^−1^ for N‐SbF_6_, N‐TFSI, and N‐ONF, respectively (*n* = 4), corresponding to a maximum increase of 132% relative to IE/DDM (Figure 4b). For reference, the DDM‐cured sample did not require a separate pre‐curing step, as its curing exotherm occurred within the LC transition temperature range (110°C–125°C) as shown in Figure S3b.

This improvement is attributed to the increased time required for molecular reorganization and maintenance of LC domain structure, leading to improved phonon transport pathway efficiency. Furthermore, the standard deviation decreased with increasing pre‐curing time, indicating improved sample‐to‐sample uniformity as well as enhanced uniformity in molecular orientation and cross‐linked network structure.

Based on this understanding of LC domain preservation, we compared naphthalene‐modified and benzyl‐based initiators under fixed 24 h pre‐curing conditions. For the same counter anion, the naphthalene‐modified initiators consistently exhibited higher thermal conductivities, demonstrating the beneficial effects of naphthalene substitution on polymerization stability and network formation. Specifically, in the SbF_6_^−^ series, B–SbF_6_ and N–SbF_6_ exhibited thermal conductivities of 0.72 ± 0.02 and 0.77 W m^−1^ K^−1^, respectively, representing approximately a 7% increase, as shown in Figure 4c. In the TFSI^−^ series, B–TFSI and N–TFSI reached 0.66 ± 0.01 and 0.86 W m^−1^ K^−1^, respectively, representing a 30% increase in Figure 4d. In the ONF^−^ series, B–ONF and N–ONF reached 0.56 ± 0.01 and 0.69 W m^−1^ K^−1^, respectively (*n* = 4), representing a 23% increase in Figure 4e.

These results are consistent with the observation in Section 2.2 that the long lifetimes and high activities of naphthalene‐modified initiators promote a denser and more homogeneous crosslinking network. The enhanced network integrity facilitates more efficient phonon transport, particularly within liquid–crystalline epoxies, and the preservation of mesophase domains contributes to enhanced thermal conductivity. Therefore, naphthalene‐modified cationic initiators exhibit superior polymerization stability and thermal conductivity compared to benzyl‐based initiators, demonstrating their effectiveness for high‐performance thermal management in epoxy thermosets.

### 2.4 X‐Ray Diffraction Analysis of Mesophase Arrangement and Its Correlation with Thermal Conductivity

To elucidate the structural origins of the differences in thermal conductivity between the composites, X‐ray diffraction (XRD) analysis was performed on IE samples cured with naphthalene‐modified and benzyl‐based cationic initiators under LC conditions.

**FIGURE 5:**
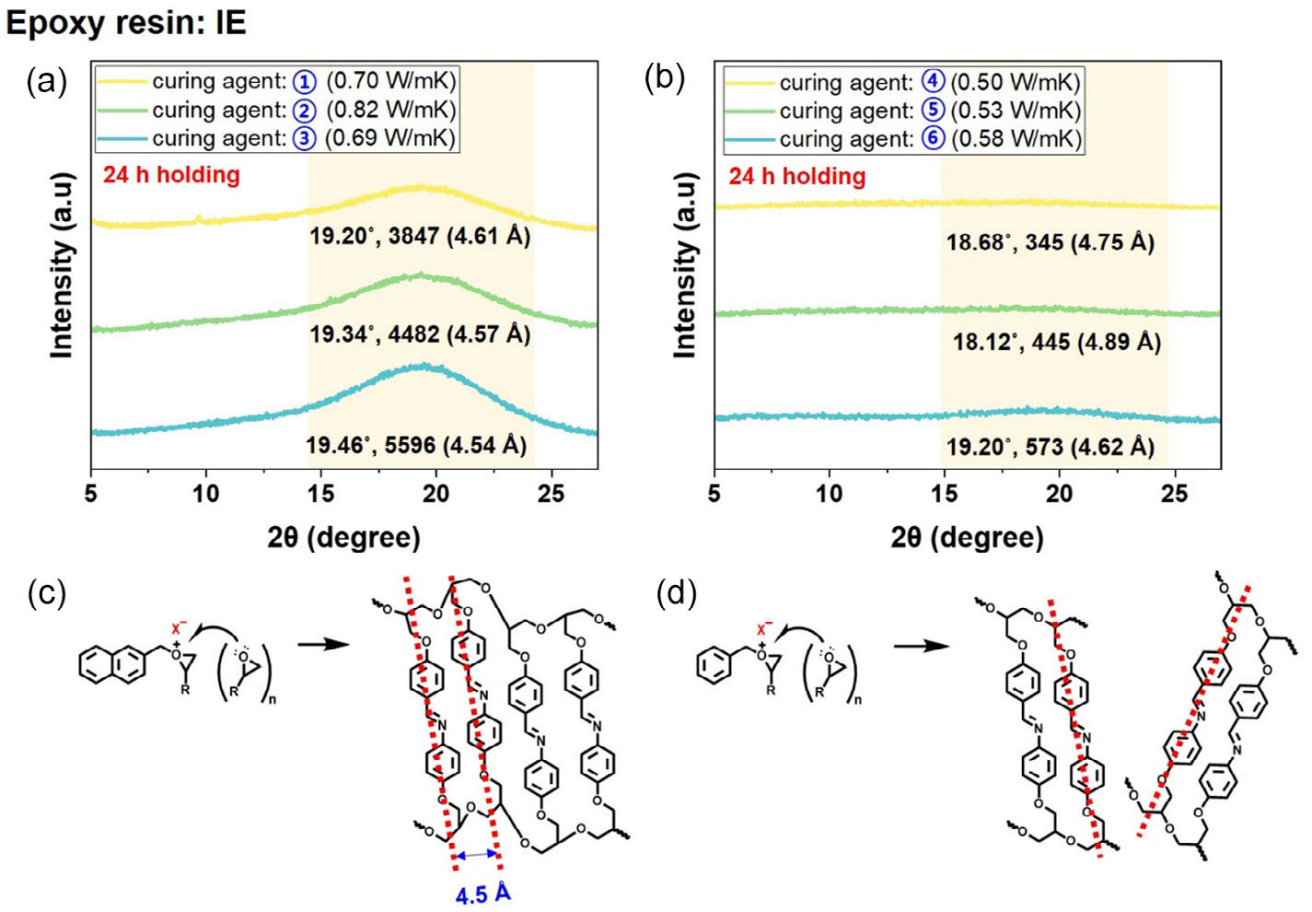
(a,b) XRD patterns of IE cured for 24 h at 120°C with (a) naphthalene‐modified and (b) benzyl‐based cationic initiators. (c,d) Schematic illustrations of polymerized IE showing molecular arrangement: (c) naphthalene‐modified initiators with well‐aligned *π*–*π* stacking at 4.5 Å (corresponding to a), and (d) benzyl‐based initiators with irregular stacking and nonuniform structure (corresponding to b). Measurements were performed at room temperature using a Rigaku SmartLab 3 kW diffractometer with Cu K*α* radiation (*λ* = 1.541 Å) at 40 kV and 30 mA, scanned over 2*θ* = 5°–90° with 0.04° step size and 5° min^−1^ scan rate.

The low‐angle diffraction peak near 2*θ* ≈ 19° corresponds to a *π–* *π* stacking distance of approximately 4.5 Å and is a key indicator of how regularly the mesogenic aromatic cores are aligned in the LC state. Therefore, the intensity and sharpness of this peak directly reflect how effectively each initiator promotes mesophase arrangement. As shown in Figure 5a, the naphthalene‐modified initiator exhibits a very high 19° peak intensity, indicating a well‐ordered structure. In contrast, the benzyl‐based initiator in Figure 5b exhibits a much lower intensity, suggesting reduced ordering. These differences are consistent with the observed thermal conductivity trends between the two series. Higher structural ordering indicates improved thermal conductivity. These observations are well consistent with the structural schematics in Figure 5c,d. Figure 5c shows that the naphthalene‐modified initiator forms a dense, regular *π* *–π* stacking structure with a spacing of approximately 4.5 Å, whereas Figure 5d shows that the benzyl‐based initiator stacks irregularly and loosely. Therefore, these structural differences visually support the XRD intensity changes at 19° and explain the higher thermal conductivity observed for the naphthalene‐modified initiator compared to the benzyl‐based initiator.

However, when comparing different counter anions for the same naphthalene or benzyl cation, the 19° peak intensity does not always directly correlate with thermal conductivity. This is because the counter anion influences the curing rate, reaction enthalpy, and the duration for which the LC domain structure is maintained during curing, all of which influence the formation of phonon transport pathways in the final network. Therefore, the 19° peak intensity provides the most reliable measure of alignment when comparing initiators with the same counteranion, whereas differences in counteranion must account for both structural alignment and dynamic curing behavior.

### 2.5 Kinetic Regulation of Mesophase Preservation and Its Impact on Thermal Conductivity

Although XRD analysis (Figure 5) provides direct evidence for preserved mesophase ordering in the cured epoxy networks, it does not explain why such ordering is maintained during the highly exothermic curing process. LC epoxy curing involves rapid network formation accompanied by significant heat release, during which excessively fast reactions can disrupt mesogen alignment due to localized thermal fluctuations and restricted molecular rearrangement. Therefore, high curing rates do not necessarily favor the preservation of LC order.

**FIGURE 6:**
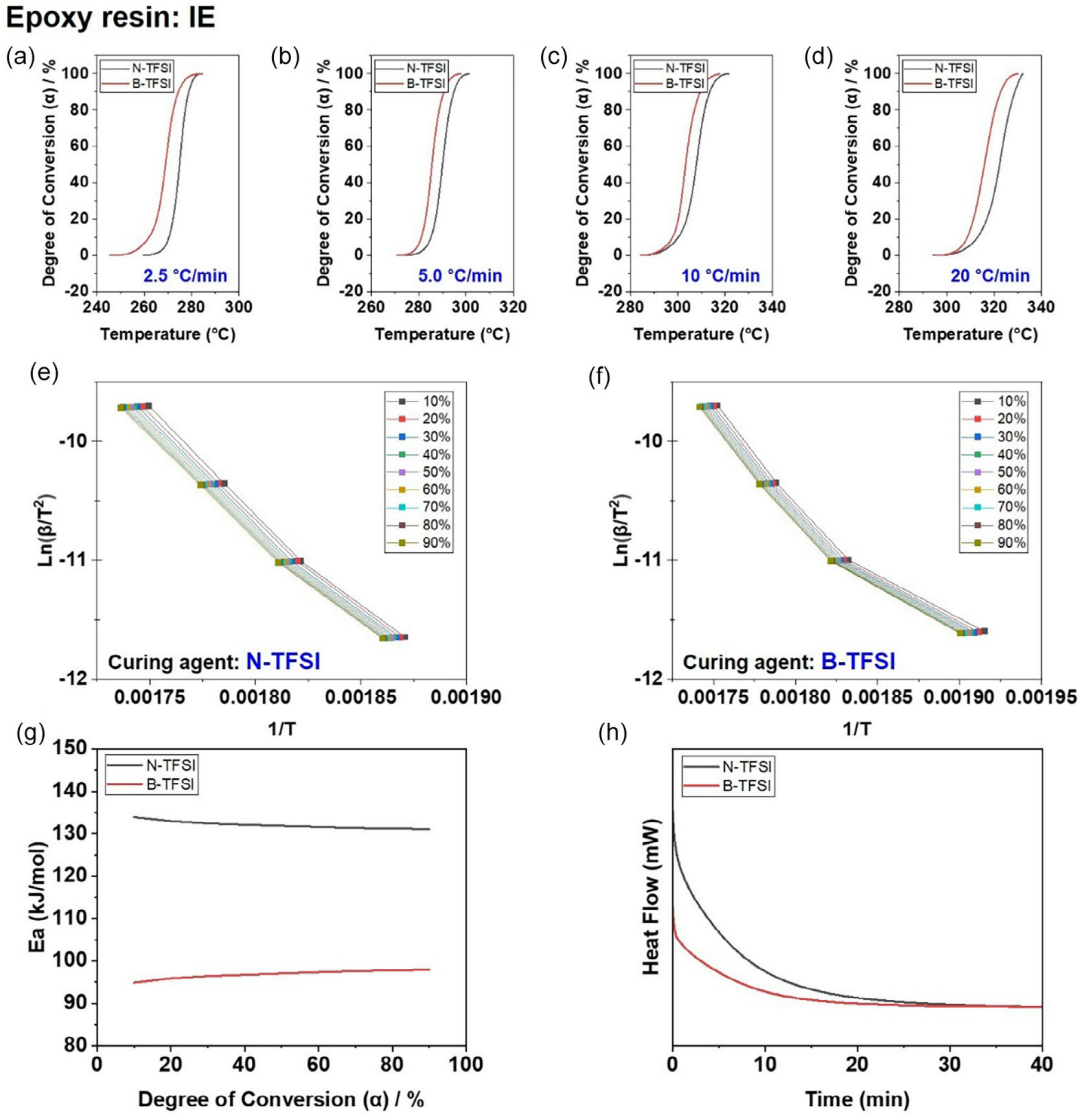
(a–d) Degree of conversion (*α*) as a function of temperature obtained from non‐isothermal DSC measurements for the imine‐based liquid crystalline epoxy (IE) cured with N‐TFSI and B‐TFSI at heating rates of 2.5, 5.0, 10, and 20°C/min, respectively, (e,f) Kissinger–Akahira–Sunose (KAS) plots of ln(*β/T^2^*) versus 1/*T* at various degrees of conversion (*α* = 10%–90%) for IE systems cured with (e) N‐TFSI and (f) B‐TFSI, (g) Activation energy (*Ea*) as a function of degree of conversion calculated using the KAS isoconversional method, (h) Isothermal DSC heat flow curves measured at 220°C, comparing the intrinsic curing behaviors and effective reaction lifetimes of the two initiator systems.

We employed kinetic analysis to elucidate the mechanism by which naphthalene‐modified initiators preserve superior LC molecular alignment compared to benzyl‐modified initiators, thereby leading to enhanced thermal conductivity. The results were interpreted by combining non‐isothermal and isothermal DSC analyses with Kissinger–Akahira–Sunose (KAS) isoconversional kinetics [15] (Figure 6). The evolution of degree of conversion (*α*) with temperature obtained from non‐isothermal DSC measurements at different heating rates (Figure 6a–d) reveals distinct curing behaviors between the two initiator systems.

Compared to the B‐TFSI system, the N‐TFSI‐cured IE exhibits a more gradual increase in *α* over a broader temperature range, particularly at lower heating rates, indicating moderated reaction kinetics and delayed network formation (Figure 6a–d). This behavior suggests that the N‐TFSI initiator enables extended molecular mobility during curing, which is favorable for mesogenic self‐organization.

The KAS plots of ln(*β*/*T* ^2^) versus 1/*T* at different conversion levels (*α* = 10%–90%) (Figure 6e,f) show good linearity for both systems, confirming the applicability of the isoconversional approach. Notably, the N‐TFSI system exhibits steeper slopes, corresponding to higher apparent activation energies, over most of the conversion range.

Accordingly, the activation energy profiles derived from the KAS analysis (Figure 6g) demonstrate that the N‐TFSI system maintains consistently higher activation energies than the B‐TFSI analog, especially at intermediate and high conversion levels. This indicates moderated reactivity and prolonged effective initiator lifetime.

The intrinsic curing behaviors were further examined by isothermal DSC measurements at 220°C (Figure 6h). The N‐TFSI system exhibits a broader and more sustained exothermic profile, whereas the B‐TFSI system shows a sharp and rapidly decaying heat flow peak. This difference reflects a more temporally distributed reaction process for N‐TFSI and a highly concentrated exothermic event for B‐TFSI.

Importantly, the moderated and temporally dispersed curing behavior induced by N‐TFSI suppresses abrupt thermal fluctuations and provides sufficient time for mesogenic units to rearrange and self‐organize during network formation. As a result, the LC domains formed during curing are more effectively preserved in the final network structure, consistent with the enhanced XRD ordering observed in Figure 5.

This kinetic–structural coupling directly contributes to the superior thermal conductivity of the N‐TFSI‐based epoxy systems. As a result of the well‐preserved mesophase ordering, the N‐TFSI‐cured composites exhibit a significantly higher thermal conductivity of 0.86 W m^−1^ K^−1^ compared to 0.66 W m^−1^ K^−1^ for the B‐TFSI‐based systems. The enhanced molecular alignment promotes efficient phonon transport through aligned aromatic domains, thereby facilitating the formation of continuous thermal conduction pathways. Consequently, initiator‐controlled curing kinetics emerge as a critical factor governing mesophase stability and thermal transport performance in LC epoxy composites. Detailed kinetic analyses, including conversion behavior, KAS isoconversional plots, activation energy profiles, and isothermal reaction persistence, are provided in Supporting Information, Section 3.12.

**FIGURE 7:**
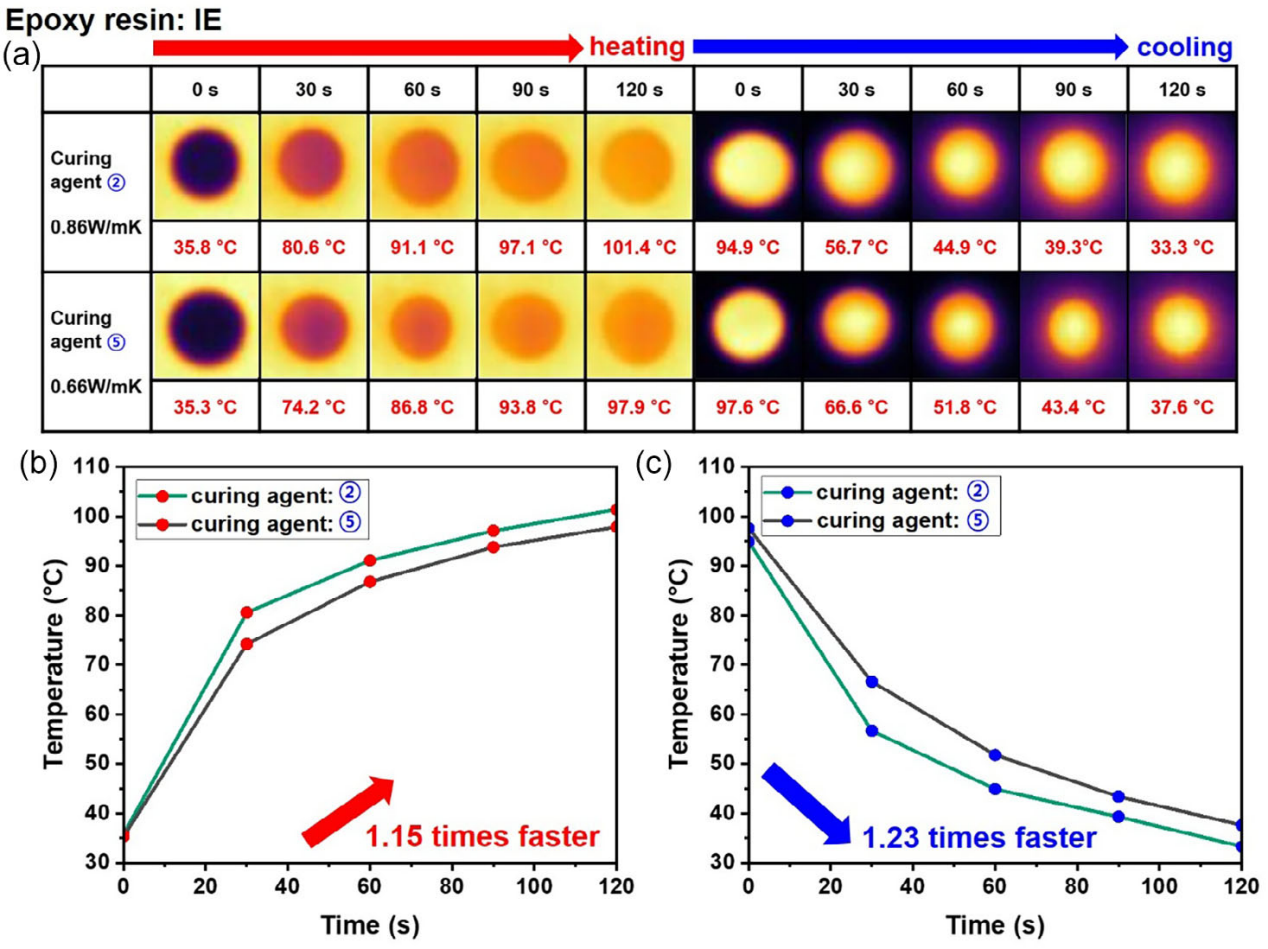
(a) Time‐lapse infrared thermal images of IE cured with naphthalene‐ and benzyl‐based cationic initiators during heating to 110°C and subsequent cooling to 30°C. (b,c) Comparison of temperature changes of IE samples with naphthalene‐ and benzyl‐based initiators: (b) heating rates, and (c) cooling rates, monitored over time using an infrared thermal camera.

To evaluate the effect of initiator structure on thermal response, time‐lapse infrared (IR) thermal images of IE samples cured with naphthalene‐ and benzyl‐based cationic initiators were recorded while heating to 110°C and cooling to 30°C (Figure 7a). Real‐time monitoring of the temperature evolution of each sample allowed for a direct comparison of their heating and cooling behaviors.

As shown in Figure 7b,c, IE samples cured with naphthalene‐modified initiators exhibited slightly faster heating and cooling rates than those cured with benzyl‐based initiators. This enhancement is attributed to the naphthalene substituents improving molecular packing and alignment within the IE network, promoting more uniform and efficient heat transfer. This difference in thermal response is consistent with the measured thermal conductivity trends, demonstrating that the structure of the cationic initiator not only influences network formation at the molecular level but also significantly impacts the macroscopic heat transfer of the cured epoxy system.

### 2.6 Mechanistic Comparison of Phonon Transport in DGEBA and Liquid Crystalline Epoxy Networks

**FIGURE 8:**
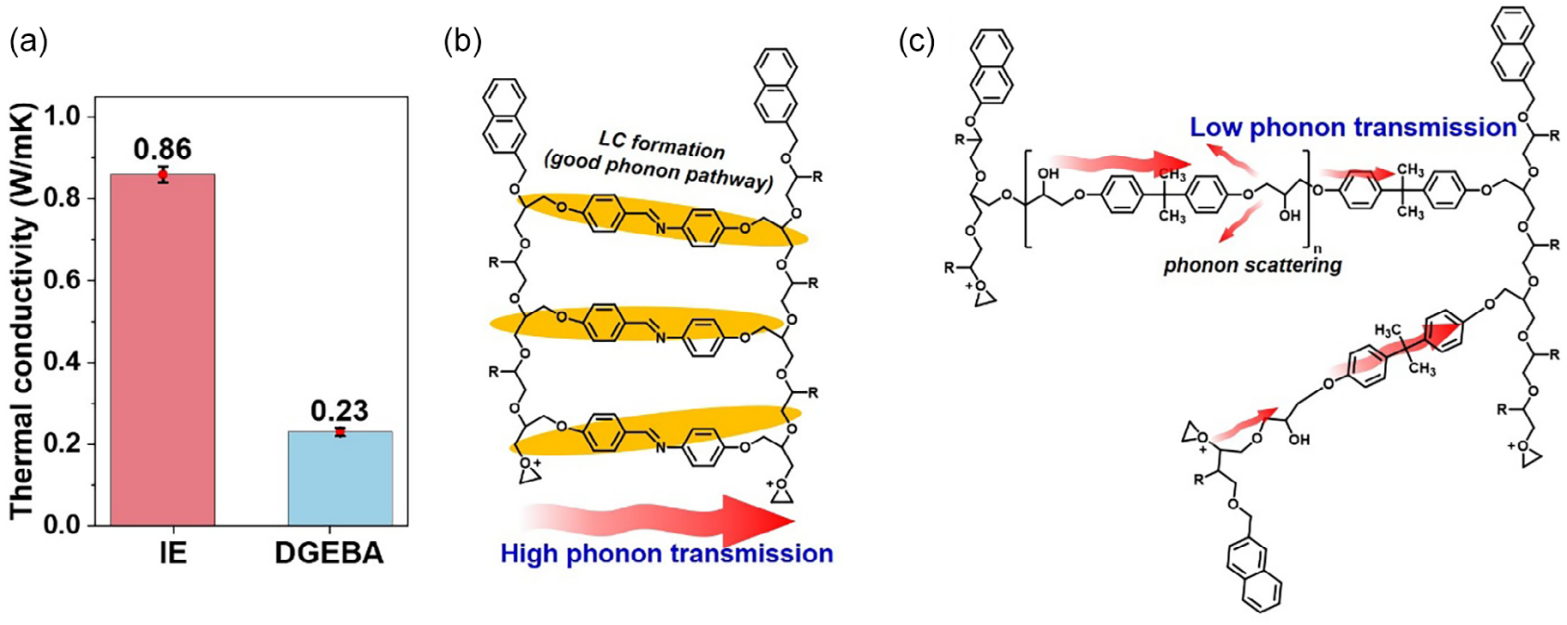
(a) Comparison of intrinsic thermal conductivity between N‐TFSI‐cured LCE and DGEBA matrices. (b) Schematic illustration of the LCE network showing the formation of locally ordered liquid crystalline domains, which provide continuous and efficient phonon transport pathways with reduced phonon scattering. (c) Schematic illustration of the DGEBA network, where the amorphous and randomly crosslinked structure induces strong phonon scattering and results in inefficient phonon transmission.

To clarify the fundamental difference in phonon transport behavior between the conventional DGEBA network and the liquid crystalline epoxy (LCE) system, a comparative analysis and schematic illustration are presented in Figure 8. As shown in Figure 8a, the intrinsic thermal conductivity of the LCE cured with the N‐TFSI initiator reaches 0.86 W m^−1^ K^−1^, which is nearly four times higher than that of the DGEBA counterpart (0.23 W m^−1^ K^−1^). This remarkable enhancement cannot be explained solely by chemical composition, but is mainly attributed to the distinct molecular packing and structural ordering in the LCE network.

As illustrated schematically in Figure 8b, the LCE forms locally ordered and partially aligned LC domains during curing, which promote continuous and less interrupted phonon transport pathways. The enhanced chain rigidity and *π*–*π* stacking of mesogenic units reduce phonon scattering and facilitate efficient phonon transmission along the ordered domains. In contrast, the DGEBA network (Figure 8c) possesses a highly amorphous and randomly crosslinked structure, where frequent phonon scattering occurs at disordered chain segments and junctions, resulting in severely hindered heat transport.

Therefore, the intrinsically higher thermal conductivity of the LCE matrix originates from its self‐organized mesogenic structure, which provides more effective phonon transport pathways even in the absence of thermally conductive fillers. This structural advantage is expected to further amplify the synergistic effect with highly conductive fillers in constructing continuous thermal conduction networks.

### 2.7 Thermal Conductivity and Structural Evolution by Vitrimer Generation

**FIGURE 9:**
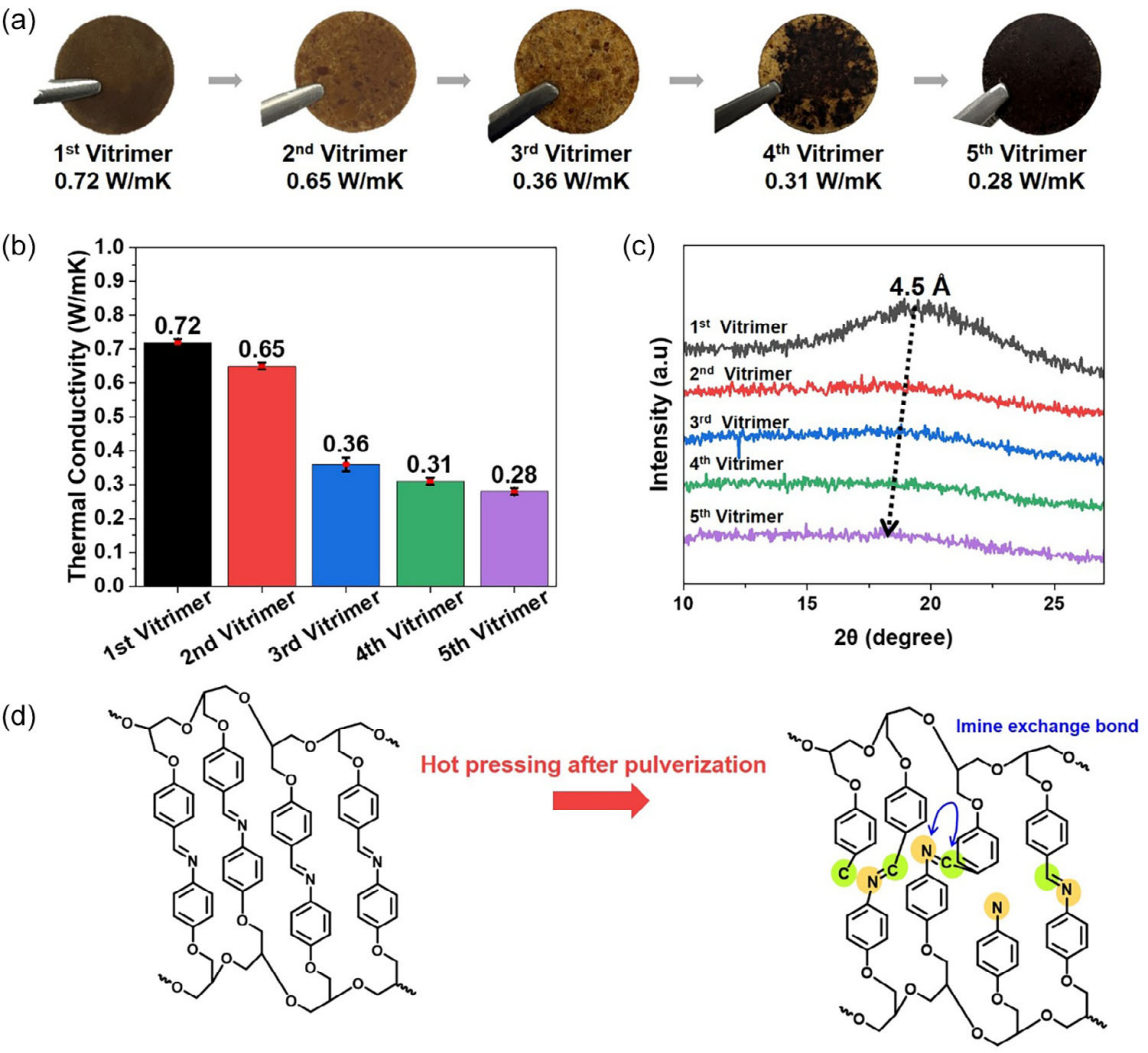
(a) Photographs of cured IE/N‐ONF vitrimer samples across successive generations; (b) comparison of in‐plane thermal conductivity; (c) XRD patterns; (d) Schematic illustration of imine bond evolution across generations, showing the first‐generation vitrimer and the sample obtained after pulverizing and hot pressing. All thermal conductivity values are presented as mean ± standard deviation obtained from four independently prepared samples (*n* = 4).

To further investigate the reprocessability and structural stability of liquid crystal epoxy systems, we designed a vitrimer containing dynamic covalent bonds. To ensure reprocessability, the vitrimer containing dynamic imine exchange bonds was synthesized using IE resin. The heat‐cured IE composite was crushed and hot‐pressed at 190°C and 30 MPa for 3 h to produce a reprocessed vitrimer sample (Figure 9a).

The first‐generation vitrimer exhibited a high thermal conductivity of 0.72 ± 0.01 W m^−1^ K^−1^, but this gradually decreased with reprocessing. The second generation had 0.65 ± 0.01 W m^−1^ K^−1^, the third generation had 0.36 ± 0.02 W m^−1^ K^−1^, the fourth generation had 0.31 ± 0.01 W m^−1^ K^−1^, and the fifth generation had 0.28 ± 0.01 W m^−1^ K^−1^ (*n* = 4) (Figure 9b). The most pronounced decrease occurred between the second and third generations, corresponding to a 45% decrease in thermal conductivity. This sharp decrease is attributed to the accumulated structural damage caused by milling during reprocessing and the lack of mesophase modification, which blocked phonon transport paths and increased interfacial scattering. XRD analysis focused on the low‐angle peak near 2*θ* ≈ 19°, indicating long‐range alignment of the mesogenic core. With each successive generation, this peak weakened significantly, shifting from 2*θ* = 20.00° in the first generation to 2*θ* = 17.76° in the fifth generation, reflecting a breakdown in lamellar order and a decrease in mesophase alignment (Figure 9c). This structural degradation closely correlates with the observed thermal conductivity trends.

A schematic diagram illustrating the evolution of dynamic imine bonding during reprocessing is presented in Figure 9d, showing the first‐generation vitrimer and the reprocessed sample obtained after pulverization and hot pressing. Despite the gradual decrease in thermal conductivity, the fifth‐generation vitrimer still maintains a thermal conductivity approximately 47% higher than that of the conventional DGEBA epoxy, highlighting the reprocessability and sustained performance of the LC‐based vitrimer network [16].

As schematically illustrated in Figure 9d, the vitrimer network is expected to undergo reversible imine bond exchange during hot pressing. To confirm that the dynamic imine bonds are chemically preserved after repeated reprocessing, FT‐IR analysis was performed for the first and fourth generation vitrimers (Figure S4). The characteristic C=N stretching band at ~1680 cm^−1^ remains clearly observable without noticeable shift or disappearance, indicating that the vitrimer network is chemically maintained and that reprocessing proceeds via dynamic bond exchange rather than irreversible degradation.

### 2.8 Superior Thermal Conductivity of Filler‐Reinforced Epoxy Composites

To evaluate the thermal conductivity of epoxy composites, an imine‐functionalized liquid crystalline epoxy (IE) was employed with N‐TFSI, the cationic initiator that exhibited the highest thermal conductivity in initial evaluation. To synchronize the LC self‐organization with network formation and to accelerate the curing process, 2E4MZ was introduced as a catalyst. DSC was conducted to assess the influence of the catalyst on curing behavior (Figure S5). The onset and peak temperatures were 125°C and 140°C, respectively, while the curing enthalpy increased from 92 J/g (without catalyst) to 195 J/g upon catalyst addition. This enhancement is attributed to the efficient proton transfer facilitated by the imidazole catalyst, which promotes faster reaction kinetics and more complete network formation.

For thermal transport optimization, hexagonal boron nitride (*h*‐BN) fillers were incorporated at loadings ranging from 60 to 90 wt%. Since the IE epoxy is a solid powder, the composites were prepared by dry blending the epoxy powder with cationic initiator (3 PHR) and fillers, followed by direct molding and curing without using any solvent (see Supporting Information, Section 3.10). During heating, the epoxy melted and cured in situ in the mold. The curing was conducted at 125°C, which lies within the LC temperature window, allowing LC self‐assembly to occur during curing. Dense composites without observable macroscopic porosity were obtained.

**FIGURE 10:**
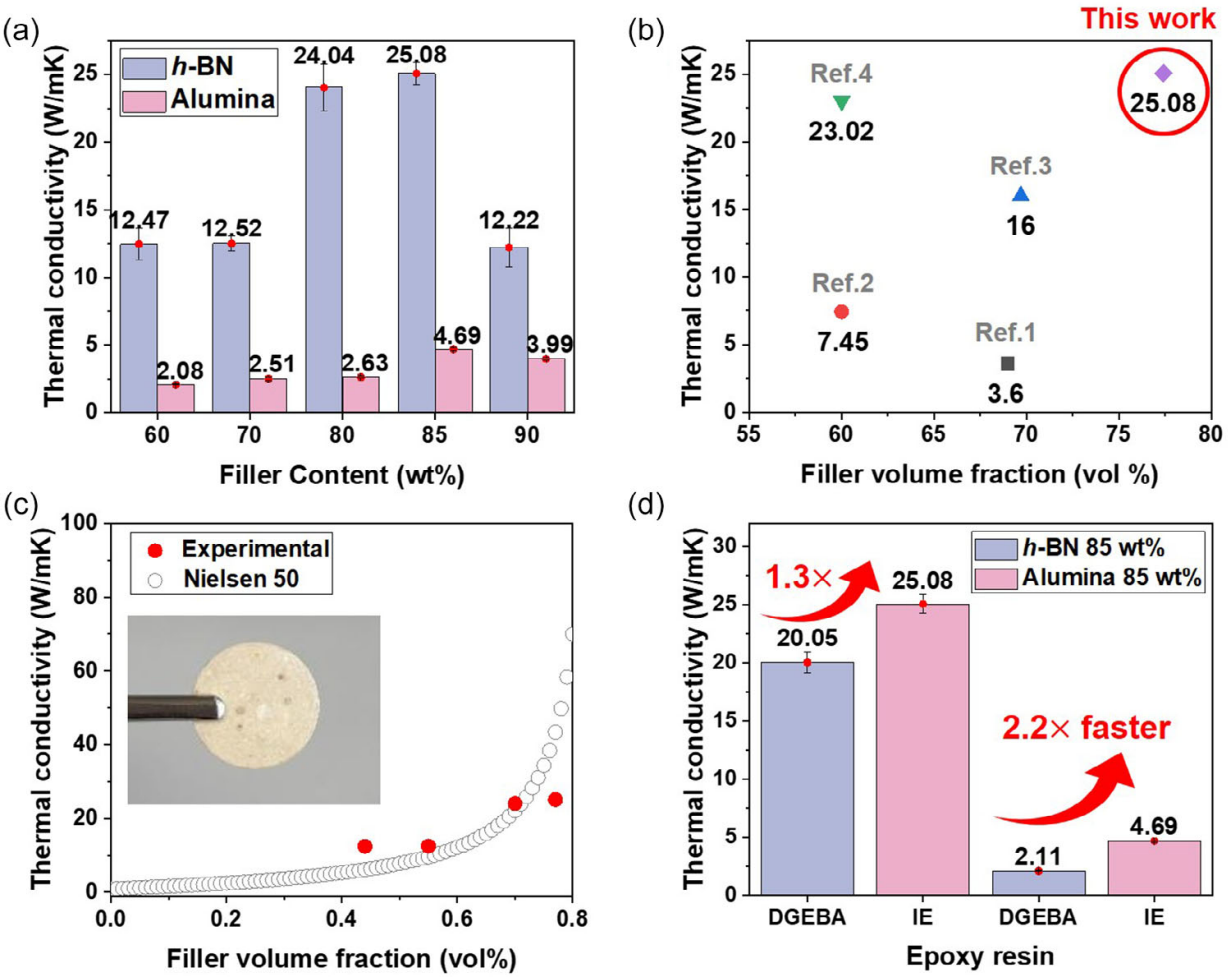
In‐plane Thermal conductivity analysis of epoxy composites cured with IE, N‐TFSI, and 2E4MZ catalyst (3 PHR). All data points represent the average of four independent measurements (*n* = 4), with error bars indicating standard deviations. (a) Comparison of thermal conductivity with varying filler loadings (60–90 wt%) for *h*‐BN and alumina, (b) comparison of experimental thermal conductivities of *h*‐BN/epoxy composites with reported literature values (Refs. [1, 2, 3, 4]), clearly demonstrating the record‐high performance of this work, (c) experimental thermal conductivities of IE/*h*‐BN composites versus the Lewis–Nielsen model predictions at different filler loading, and (d) comparison of thermal conductivities between IE‐ and DGEBA‐based composites at 85 wt% *h*‐BN and alumina.

The measured thermal conductivities were 12.47 ± 1.15, 12.52 ± 0.55, 24.04 ± 1.75, 25.08 ± 0.79, and 12.22 ± 1.41 W m^−1^ K^−1^, respectively (*n* = 4) (Figure 10a). Notably, at 85 wt% *h*‐BN, the thermal conductivity reached 25.08 W m^−1^ K^−1^. Previously reported thermal conductivities of epoxy composites using *h*‐BN fillers ranged from 3.6 to 23.02 W m^−1^ K^−1^ [4, 8, 17, 18], whereas our experiment achieved a higher value of 25.08 W m^−1^ K^−1^, surpassing the previously reported maximum (Figure 10b).

At 90 wt%, however, the thermal conductivity decreased to 12.22 W m^−1^ K^−1^, which is attributed to the excessively high filler loading that results in insufficient epoxy infiltration, poor packing density, and degraded interparticle thermal contact, thereby disrupting the formation of continuous thermal pathways. This interpretation is further supported by the photographic images of the composites (Figure S7), where the specimen containing 90 wt% *h*‐BN exhibits severe brittleness and partial structural collapse during demolding and handling, indicating a loss of matrix continuity at excessively high filler loading.

To further elucidate the structure–property relationship of the filler‐reinforced epoxy composites, the experimental thermal conductivity data were systematically analyzed using multiple theoretical models, including the Lewis–Nielsen model with aspect ratios of *p* = 30 and 50 and the Agari–Uno model (Supporting Information, Section 3.11). These models represent different approaches to describing thermal transport in composite systems. The Lewis–Nielsen model explicitly incorporates filler geometry and orientation effects through the aspect ratio parameter, making it suitable for anisotropic platelet fillers such as *h*‐BN, whereas the Agari–Uno model is a semi‐empirical formulation that does not explicitly consider filler shape and is mainly used to describe percolation‐driven thermal transport behavior.

As shown in Figures 10c and S6, both Lewis–Nielsen models with *p* = 30 and 50 successfully reproduce the overall increasing trend of thermal conductivity with filler loading for the *h*‐BN‐filled composites. However, the model with *p* = 50 exhibits consistently better agreement with the experimental data, particularly at high filler contents, indicating more effective representation of the platelet geometry and partial alignment of *h*‐BN within the LC epoxy matrix. This result suggests that the LC‐induced molecular ordering facilitates the formation of continuous and anisotropic thermal transport pathways. Therefore, *p* = 50 was adopted as the most representative effective aspect ratio for the present composite system.

In contrast, the Agari–Uno model shows larger deviations from the experimental data at high filler loadings, reflecting its limited capability to capture anisotropic heat transport and alignment effects in LC systems. These results demonstrate that explicit consideration of filler geometry and orientation is essential for accurately describing thermal transport in the present *h*‐BN‐filled composites.

A similar modeling analysis was also performed for the Al_2_O_3_‐filled composites using the same theoretical frameworks (Supporting Information, Section 3.11). For the nearly spherical alumina particles, the Lewis–Nielsen model with a low aspect ratio (*p* = 1.2) showed noticeable deviation from the experimental data, whereas the Agari–Uno model provided better overall agreement across the investigated filler content range. This behavior reflects the nearly isotropic geometry of alumina fillers, for which heat transport is dominated by percolation and interparticle contact rather than directional alignment. Consequently, semi‐empirical models without explicit shape factors are more suitable for describing thermal transport in such particulate‐filled systems.

Together, these comparative modeling results highlight that the dominant heat transport mechanism in the present composites strongly depends on filler geometry and matrix‐induced alignment. Platelet‐shaped *h*‐BN fillers benefit from LC‐assisted orientation and are best described by geometry‐sensitive models, whereas nearly isotropic alumina fillers follow percolation‐dominated transport behavior. This clear distinction further supports the structural origin of the superior thermal conductivity achieved in the present LC epoxy‐based composites.

Parallel experiments were conducted using 20 µm alumina fillers under the same conditions. Thermal conductivity increased gradually from 2.08 ± 0.04 W m^−1^ K^−1^ at 60 wt% to 4.69 ± 0.08 W m^−1^ K^−1^ at 85 wt% (*n* = 4). Although the absolute thermal conductivity of alumina composites is considerably lower than that of *h*‐BN, it is higher than that of composites using conventional curing agents with 90 wt% alumina, indicating that high filler loadings can effectively establish stable thermal pathways (Figure 10a).

Finally, to evaluate the universality of this approach, all fillers were fixed at 85 wt% and the same experimental protocol was applied to DGEBA epoxy. The DGEBA composite with 85 wt% *h*‐BN exhibited a thermal conductivity of 20.05 ± 0.93 W m^−1^ K^−1^, whereas the composite with 85 wt% alumina showed only 2.11 ± 0.03 W m^−1^ K^−1^ (*n* = 4) (Figure 10d). Moreover, the IE system demonstrated even higher thermal conductivity compared to conventional DGEBA, underscoring the effectiveness of molecular alignment in facilitating phonon transport as previously observed in LC epoxy/BN composites [19]. Notably, even for standard DGEBA, the incorporation of the naphthalene‐modified cationic initiator led to significantly enhanced thermal conductivity compared to conventional curing agents, confirming the broad applicability of this strategy.

## 3 Conclusion

This study demonstrates that the rational design of a naphthalene‐modified cationic initiator with tunable counteranions effectively extends the working lifetime of epoxy curing, enabling efficient polymerization and maintenance of an intrinsic ordered structure in both conventional and liquid–crystalline (LC) epoxy thermosets. The prolonged activity of these initiators ensures that the LC domains maintain order during network formation, resulting in an unfilled LC epoxy with an exceptionally high thermal conductivity of 0.86 W m^−1^ K^−1^, a very high value for a purely organic system. Compared to benzyl‐based initiators, the naphthalene‐modified counterpart consistently produces a denser network and enhanced heat transfer. The addition of thermally conductive fillers further enhances thermal conductivity, with 85 wt% *h*‐BN composites achieving a thermal conductivity exceeding 25 W m^−1^ K^−1^, outperforming alumina‐filled systems with similar filler content. Even in conventional DGEBA matrices, the initiator strategy significantly enhanced the filler effect. Moreover, the reprocessable vitrimer system retained a significant portion of its thermal performance even after multiple recycling cycles, demonstrating structural adaptability through a dynamic covalent bonding network.

These results demonstrate that precise control over initiator chemistry and curing kinetics can be utilized to construct highly ordered, thermally conductive, and recyclable epoxy networks. This study not only provides fundamental insights into the structure–property relationships of thermosets but also offers a versatile platform for next‐generation thermal management materials, electronics, and energy‐efficient composites.

## 4 Experimental Section

4.1

All experimental details are provided in the Supporting Information.

## Supporting Information

Additional supporting information can be found online in the Supporting Information section. **Supporting Fig. S1:** (a) ^1^H NMR spectrum of naphthalene‐modified cationic initiator, (b) ^1^H NMR spectrum of benzyl‐based cationic initiator, and (c) ^1^H NMR spectrum of the synthesized imine epoxy (IE) ^1^H NMR spectra were acquired at 500 MHz using a JEOL 500 spectrometer (Japan) with DMSO‐d_6_ as the solvent. Pulse width: 1 μs; relaxation delay: 2 s; 32 scans per sample. **Supporting Fig. S2:** (a‐c) DSC first heating and cooling curves of DGEBA epoxy systems containing each benzyl‐based cationic initiator, measured under a nitrogen atmosphere at a heating/cooling rate of 10 °C·min^‐1^ (a) Benzyl‐based hexafluoroantimonate (B‐SbF_6_), (b) Benzyl‐based bis(trifluoromethanesulfonyl)imide (B‐TFSI), (c) Benzyl‐based nonafluoro‐1‐butanesulfonate (B‐ONF). (d‐f) Storage and loss modulus as a function of time for DGEBA epoxy systems with each initiator, measured while increasing the temperature at a rate of 3 °C ·min^‐1^ (d) B‐SbF_6_, (e) B‐TFSI, and (f) B‐ONF. **Supporting Fig. S3:** DSC first heating and cooling curves of (a) DGEBA/MeTHPA and (b) IE/DDM systems measured under nitrogen atmosphere at a heating rate of 10 °C min^−1^. The thermal conductivity values determined for (a) and (b) were 0.1940 and 0.3700 W/m·K, respectively. **Supporting Fig. S4:** FT‐IR spectra of the first‐ and fourth‐generation vitrimer samples after repeated reprocessing cycles. The characteristic C=N stretching vibration (~1680 cm^−1^) is clearly preserved with negligible change in both intensity and position, confirming that the imine‐linked network structure is chemically maintained during hot‐press reprocessing. **Supporting Fig. S5:** DSC first heating and cooling curves of (a) IE/N‐TFSI (3 PHR) and (b) IE/N‐TFSI (3 PHR)/2E4MZ (3 PHR) systems measured under nitrogen atmosphere at a heating rate of 10 °C·min^−^¹, showing the effect of the catalyst in curing behavior. **Supporting Fig. S6:** Comparison between experimental thermal conductivity values and model predictions for (a–c) IE/*h*‐BN composites and (d,e) IE/Al_2_O_3_ composites. (a) Lewis–Nielsen model with *P* = 30 for *h*‐BN‐filled composites, (b) Lewis–Nielsen model with *P* = 50 for *h*‐BN‐filled composites, (c) Agari–Uno model for *h*‐BN‐filled composites, (d) Lewis–Nielsen model with *P* = 1.2 for Al_2_O_3_‐filled composites, and (e) Agari–Uno model for Al_2_O_3_‐filled composites. **Supporting Fig. S7:** Photographs of the cured IE/*h*‐BN composites containing (a) 60 wt%, (b) 70 wt%, (c) 80 wt%, (d) 85 wt%, and (e) 90 wt% *h*‐BN filler. The corresponding thermal conductivity values are 12.47, 12.52, 24.04, 25.08, and 12.22 W m^−1^ K^−1^, respectively. The composite with 90 wt% *h*‐BN exhibits severe brittleness and partial structural collapse during demolding and handling, indicating insufficient matrix continuity at excessively high filler loading.

## Conflicts of Interest

The authors declare no conflicts of interest.

## Supporting information

Supplementary Material

## Data Availability

The data that support the findings of this study are available in the supplementary material of this article.
