# Supplementary material for: Naphthalene‐Modified Cationic Initiators for Superior Polymerization Stability and Thermal Conductivity in Epoxy Thermosets
Source: Small Sci. 2026 Mar 6;6(3):e202500625. doi: 10.1002/smsc.202500625 (PMC12970198; doi:10.1002/smsc.202500625)
Supplement: Supplementary file 1 — Supplementary Material [file SMSC-6-e202500625-s001.pdf]

Supporting Information

Naphthalene-Modified Cationic Initiators for Superior Polymerization Stability and Thermal Conductivity in Epoxy Thermosets

Yewon Woo [a], *Yeonha Ju*[a], *Naye Hong*[a], *Kyeong Pang*[b], *Mooho Lee*[b], *In Kim*[b], *Munju Goh* [a]*

[a] Department of Chemical Engineering, Konkuk University, 120 Neungdong–ro, Gwangjin–gu, Seoul 05029, Republic of Korea

[b] Material Research Center, Samsung Advanced Institute of Technology, Samsung Electronics Co., Ltd, Suwon, Gyeonggi-do 16678, Republic of Korea

*Corresponding author's E-mail address: mgoh@konkuk.ac.kr

Table of Contents

1. Materials and reagents 3
2. General information 3
2.1 Proton nuclear magnetic resonance spectroscopy (^{1}H NMR) 3
2.2 Differential scanning calorimetry (DSC) 3
2.3 Dynamic mechanical analyses (DMA) 3
2.4 Hot Disk TPS 2500 3
2.5 X-Ray Diffraction (XRD) 4
3. Experiment methods section 4
3.1 Synthesis of naphthalene-modified cationic initiator 4
3.2 Synthesis of benzyl based cationic initiator 4
3.3 Procedure for anion exchange 4
3.4 Synthesis of imine-functionalized liquid crystalline epoxy (IE) 5
3.5 Procedure for curing DGEBA with three different naphthalene-modified cationic initiators 6
3.6 Procedure for curing IE with three different benzyl-based cationic initiators 6
3.7 Procedure for curing IE with three different naphthalene-modified cationic initiators 6
3.8 Procedure for curing DGEBA and IE resins with conventional curing agents 7
3.9 FT-IR Analysis of Vitrimer Reprocessing Cycles 7
3.10 Procedure for curing IE composites with filler 8
3.11 Comparison of theoretical models for thermal conductivity prediction 9
3.12 Kinetic analysis of curing behavior 12
4. References 13

1. Materials and reagents
4-Hydroxybenzaldehyde (98%), 4-aminophenyl (98%), N,N,N',N'-tetramethylpheylene-1,4-diamine (MeTHPA), and 4,4′-diaminodiphenylmethane (DDM, 97.0%), were purchased from Sigma-Aldrich (USA). Epichlorohydrin (99%) and silver nitrate (99.5-100%) were obtained from Daejung Chemicals & Metals Co. (Korea). Tetrabutylammonium bromide (TBAB, 98%), pyrazine (98%) and 2-Ethyl-4-Methylimidazole (95%) were purchased from Tokyo Chemical Industry Co. (TCI, Japan). Sodium hydroxide (NaOH, 98%) and acetonitrile (99.5%) were obtained from Samchun Pure Chemical Co. and Duksan (Korea), respectively. 2-(Bromomethyl)naphthalene (96%), sodium hexafluiri-1-butanesulfonate (98%), potassium nonafluoro-1-butanesulfonate(98%) and lithium bis(trifluoromethanesulfonyl)imide were supplied by Sigma-Aldrich (USA). Ethanol (EtOH), methanol (MeOH), ethyl acetate (EA), dichloromethane (DCM), petroleum ether, and sodium sulfate anhydrous (99%) were obtained from Duksan (Korea) and used as received. Hexagonal boron nitride (*h*-BN) was purchased from Denka Company Limited (Japan), and 20 μm alumina (Al2O_3) was kindly provided by the Material Research Center, Samsung Advanced Institute of Technology, Samsung Electroniccs (Korea).

2. General information
2.1 Proton nuclear magnetic resonance spectroscopy (^{1}H NMR)

^{1}H NMR analysis of synthesized molecules was carried out at 500 MHz on a JEOL 500 spectrometer (Japan) using DMSO-d6 as the solvent. The pulse width was 1 μs, the delay time was 2 s and 32 scans per sample dissolved in DMSO-d6 and packed into a glass sample tube were coated. (solvent DMSO-d_6: 2.49 for ^{1}H, s: singlet, d: doublet, t: triplet, q: quartet, and m: multiplet).

2.2 Differential scanning calorimetry (DSC)

The discovery DSC 25 was used to perform DSC under a nitrogen atmosphere. 3-4 mg of IE and DGEBA epoxy and 3 PHR of cationic initiator were encapsulated in an aluminum crucible and placed in the machine before the measurements. Each material was measured at a heating and cooling rate of 10 °C/min.

2.3 Dynamic mechanical analyses (DMA)

The DMA850 Dynamic Mechanical Analyzer (TA Instruments, New Castle, DE, USA) was used to measure the dynamic mechanical properties of DGEBA /cationic initiator in this experiment. Measurements were uniformly 35 mm (length)×13 mm (width) 3 mm (thickness) in size. It was scanned with a heating rate of 3 °C/min from 30 °C to 200 °C with an amplitude of 10 μm and a frequency of 1 Hz in a double cantilever mode.

2.4 Hot Disk TPS 2500

Hot Disk TPS 2500 thermal constants analyzer (ThermTest Inc. Sweden) was used to measure in the isotropic mode the thermal conductivity and thermal diffusivity according to the transient plane source method. The frequency is set to 60 Hz, the heating power is set from 10 mW to 500 mW and the measurement time is set to each specimen from 1 s to 10 s. the thermal conductivity of cured disk-shaped composite was measured in isotropic modes. All thermal conductivity values reported in this work correspond to the in-plane direction

2.5 X-Ray Diffraction (XRD)

XRD patterns were collected at room temperature using a Rigaku SmartLab 3 kW X-ray diffractometer (Rigaku, Japan) equipped with a copper target X-ray source (Cu Kα, λ = 1.541 Å) operating at 40 kV and 30 mA. Samples were scanned over 2θ range of 5- 90° with a step size of 0.04° and a scan rate of 5°/min.

3. Experiment methods section
3.1 Synthesis of naphthalene-modified cationic initiator

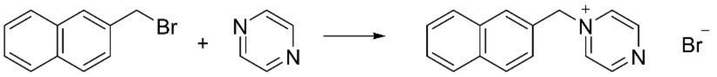

Bromonaphthalene pyrazinium was synthesized by stirring pyrazine (3.0 g, 37.46 mmol) and 2-(bromomethyl)naphthalene (6.5 g, 37.46 mmol) in 50 mL of acetonitrile at room temperature for 24 h. The resulting solid was collected by vacuum filtration, washed with appropriate solvents to remove impurities, and dried under vacuum 95% yield.
The chemical structure of the product was confirmed by ^{1}H NMR spectroscopy (500 HMz, DMSO-d6): δ (ppm) = 6.08(s, 2H), 7.55(m, J=9.74 Hz , 2H), 7.65(q, J=8.59 Hz , 1H), 7.92(m, 2H), 7.98(d, J=8.59 Hz , 1H), 8.14(s, 1H), 9.35(s, 2H), 9.54(s, 2H) **(figure S1b)**

3.2 Synthesis of benzyl based cationic initiator

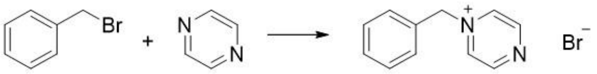

Benzyl bromide pyrazinium was synthesized by stirring pyrazine (3.0 g, 37.46 mmol) and benzyl bromide (6.4 g, 37.46 mmol) in 50 mL of acetonitrile at room temperature for 24 h. The resulting solid was collected by vacuum filtration, washed with appropriate solvents to remove impurities, and dried under vacuum 95% yield.
The chemical structure of the product was confirmed by ^{1}H NMR spectroscopy (500 HMz, DMSO-d6): δ (ppm) = 5.87(s, 2H), 7.43(q, 3H), 7.56(m, 2H**)**, 9.26(s, 2H), 9.51(s, 2H) **(figure S1a)**

3.3 Procedure for anion exchange

Anion exchange of bromonaphthalene pyrazinium was carried out with three different anions: sodium hexafluoroantimonate, potassium nonafluoro-1-butanesulfonate, and lithium bis(trifluoromethanesulfonyl)imide. Bromonaphthalene pyrazinium (3.0 g, 9.96 mmol) was dissolved in distilled water, and each anion was added at a 1:1.2 molar ratio (pyrazinium:anion) to ensure complete ion exchange. (The amounts of each anion added are as follows: $NaSbF_6$: 3.09 g, 11.95 mmol, KONF: 4.04 g, 11.95 mmol, LiTFSI: 3.43 g, 11.95 mmol.) Each mixture was stirred at room temperature for 10 min. For the $NaSbF_6$ and KONF exchanges, the product was isolated by filtration or precipitation, followed by washing and drying under vacuum.

For the LiTFSI exchanges, due to its ionic liquid nature, stirring led to phase separation and precipitation of a liquid phase. The mixture was dissolved in dichloromethane (DCM) and subjected to liquid-liquid extraction using a separation funnel to separate the organic and aqueous layers. The organic phase was collected and evaporated using a rotary evaporator to obtain the anion-exchanged product.[2]

Procedure for conformation of anion exchange

To verify the success of anion exchange, a qualitative test was performed. The exchanged product was dissolved in distilled water and 1 mL of 0.01 M silver nitrate ($AgNO_3$) solution were added. The formation of turbidity or a white precipitate indicated residual bromide ions, suggesting incomplete exchange. Conversely, a clear and transparent solution conformed effective replacement of bromide ions by the desired anions.[2] (**Figure S1c**)

3.4 Synthesis of imine-functionalized liquid crystalline epoxy (IE)

The IE was synthesized through a two-step procedure as follows:

Step 1: Synthesis of IE Precursor

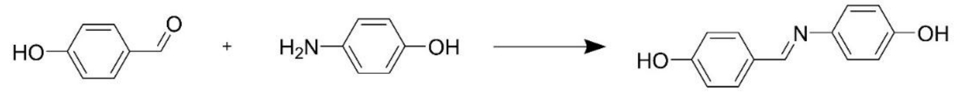

4-Hydroxybenzaldehyde 4-Aminophenyl Precursor

4-Hydroxybenzaldehyde (3.0 g, 24.57 mmol) and 4-aminophenol (2.68 g, 24.57 mmol) were dissolved in 40 mL ethanol (EtOH) and stirred at 70 °C for 90 min. After completion of the reaction, the mixture was cooled to room temperature, followed by successive washings with ethyl acetate (EA) and distilled water. The resulting organic layer was separated, dried over anhydrous sodium sulfate, and the solvent was removed using rotary evaporation. The obtained solid was dried under vacuum at 60 °C overnight, yielding the IE precursor.[1]

Step 2: Synthesis of IE

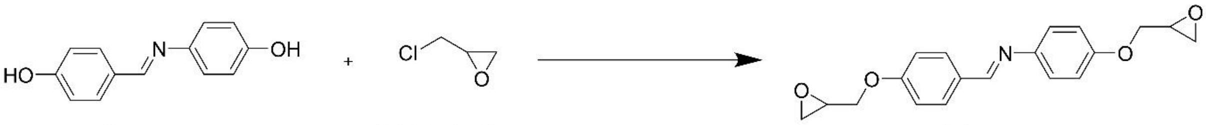

Precursor Epichlorohydrin Imine epoxy

The precursor (3.0 g, 14.4 mmol) was dissolved in an excess amount of epichlorohydrin (50mL) and tetrabutylammonium bromide (TBAB) (1.5 g, 4.6 mmol) was added as a phase transfer catalyst. The reaction mixture was stirred at 85 °C for 90 min. Subsequently, a 30 wt% aqueous NaOH solution was added dropwise under continuous stirring, and the reaction was allowed to proceed for an additional 3 h in an ice bath. The crude product was obtained by recrystallization with petroleum ether, followed by washing with methanol (MeOH). The solid was filtered and further dried under vacuum at 60 °C overnight, affording the final IE compound in 75% yield.[1]

The chemical structure of the product was confirmed by ^1H NMR spectroscopy (500 HMz, DMSO-d6): δ (ppm) = 2.69(m, J=16.04 Hz, 2H), 2.82(m, J=16.04 Hz, 2H), 3.31(m,1H), 3.84(m, J=14.32 Hz, 2H), 4.34(m, J=14.32 Hz, 2H), 6.95(m, J=8.59 Hz , 2H), 7.04(m, J=6.87 Hz , 2H), 7.20(m, J=12.03 Hz , 2H), 7.82(d, J=9.16 Hz, 2H), 8.51(s, 1H) **(figure S1c)**

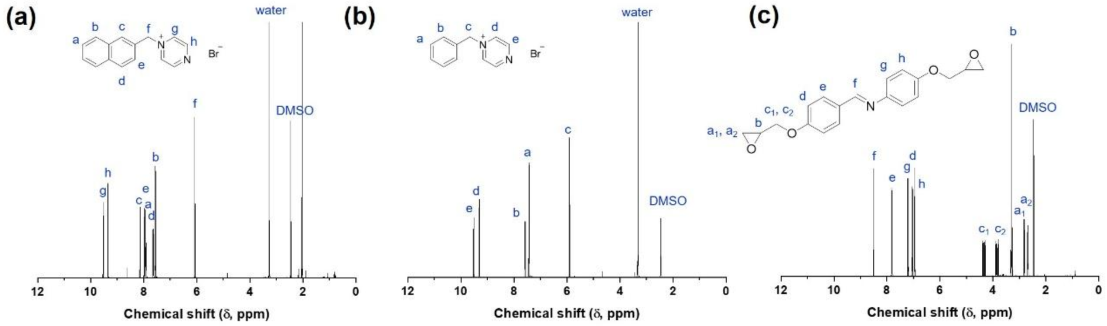

Figure S1. (a) ^1H NMR spectrum of naphthalene-modified cationic initiator, (b) ^1H NMR spectrum of benzyl-based cationic initiator, and (c) ^1H NMR spectrum of the synthesized

imine epoxy (IE) ^{1}H NMR spectra were acquired at 500 MHz using a JEOL 500 spectrometer (Japan) with DMSO-d_6 as the solvent. Pulse width: 1 μs; relaxation delay: 2 s; 32 scans per sample.

3.5 Procedure for curing DGEBA with three different naphthalene-modified cationic initiators

The curing behavior of the epoxy formulations was evaluated using differential scanning calorimetry (DSC). DGEBA resin containing 3 parts per hundred resin (PHR) of the synthesized cationic initiator was thoroughly mixed using a paste mixer (KURABO). The resulting homogeneous mixture was transferred into a hot-press mold and cured under 5 MPa pressure for 3 h at the exothermic peak temperature determined by DSC analysis. Upon completion of curing, the samples were cooled to room temperature, demolded, and polished to obtain the final cured specimens.

3.6 Procedure for curing IE with three different benzyl-based cationic initiators

The curing of DGEBA with three different benzyl-modified cationic initiators (B-SbF6, B-TFSI, and B-ONF) was performed using the same procedure as described in Section 3.5 with the corresponding DSC-determinded exothermic peak temperatures **(Figure S2)**

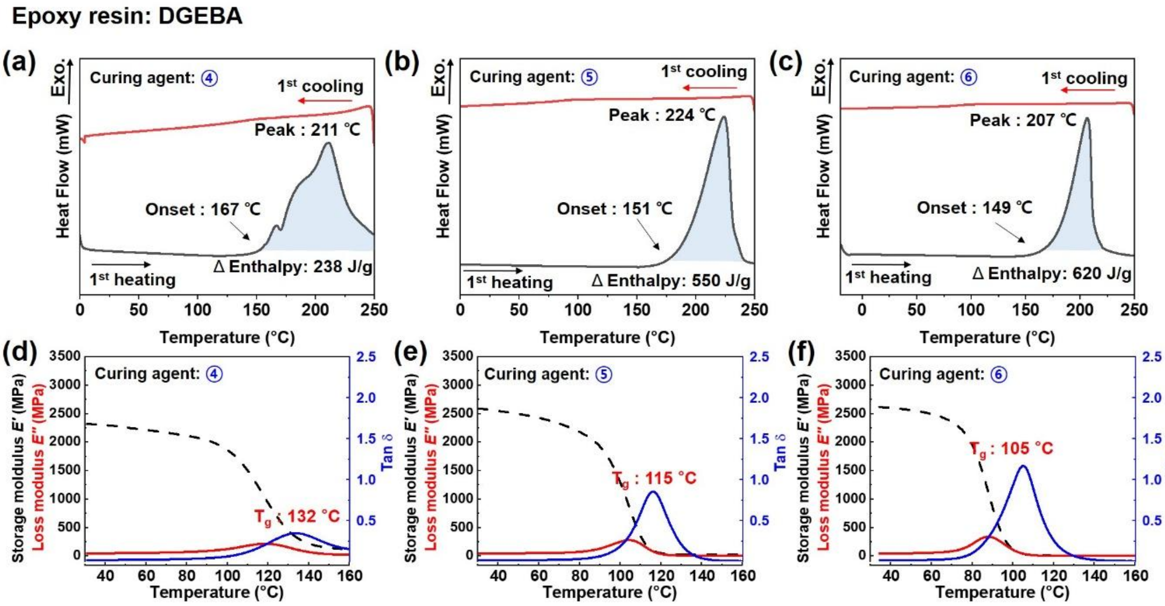

Figure S2. (a-c) DSC first heating and cooling curves of DGEBA epoxy systems containing each benzyl-based cationic initiator, measured under a nitrogen atmosphere at a heating/cooling rate of 10 °C·min^{-1} (a) Benzyl-based hexafluoroantimonate (B-SbF_6), (b) Benzyl-based bis(trifluoromethanesulfonyl)imide (B-TFSI), (c) Benzyl-based nonafluoro-1-butanesulfonate (B-ONF). (d-f) Storage and loss modulus as a function of time for DGEBA epoxy systems with each initiator, measured while increasing the temperature at a rate of 3 °C ·min^{-1} (d) B-SbF_6, (e) B-TFSI, and (f) B-ONF.

3.7 Procedure for curing IE with three different naphthalene-modified cationic initiators

For IE formulations, the same mixing procedure was employed with 3 PHR of cationic initiator. Given the critical importance of achieving successful curing within the temperature window corresponding to liquid crystalline phase formation, a two-step curing protocol was implemented to preserve the mesogenic alignment characteristic of the IE's liquid crystalline

phase. This protocol involved a re-curing step at 120 °C for 24 h, within the liquid crystalline temperature range, followed by a post-curing step at 180 °C for 3 h. This approach effectively maintained mesophase alignment throughout the curing process, yielding cured samples with enhanced molecular order.

3.8 Procedure for curing DGEBA and IE resins with conventional curing agents

The curing behavior of DGEBA and IE formulations with conventional amine-based hardeners was evaluated using differential scanning calorimetry (DSC). DGEBA resin containing N,N,N', N'-tetramethylpheylene-1,4-diamine (MeTHPA) and IE resin containing 4,4'-diaminodiphenylmethane (DDM) were thoroughly mixed at an epoxy-to-amine curing agent molar ratio of 2:1 using a paste mixer (KURABO) to obtain homogeneous mixtures. For the DDM-cured IE samples, no-separate pre-curing step ws required, as the curing exotherm occurred within the liquid crystal (LC) transition temperature range.

The homogeneous mixtures were transferred into a hot-press mold and cured under 5 Mpa pressure for 3 h at the exothermic peak temperature determined by DSC analysis. Upon completion of curing, the samples were cooled to room temperature, demolded, and polished to obtain the final cured specimens.

DSC analysis was performed to characterize the curing behavior. **Figure S3** shows the first heating and cooling curves measured under a nitrogen atmosphere at a heating rate of 10 °C min^{-1}

(a) DGEBA/ MeTHPA – thermal conductivity 0.1940 W/m·K
(b) IE/DDM – thermal conductivity 0.3700 W/m·K

These procedures provided well-cured control samples suitable for comparison with those prepared using the thermally stable cationic initiators.

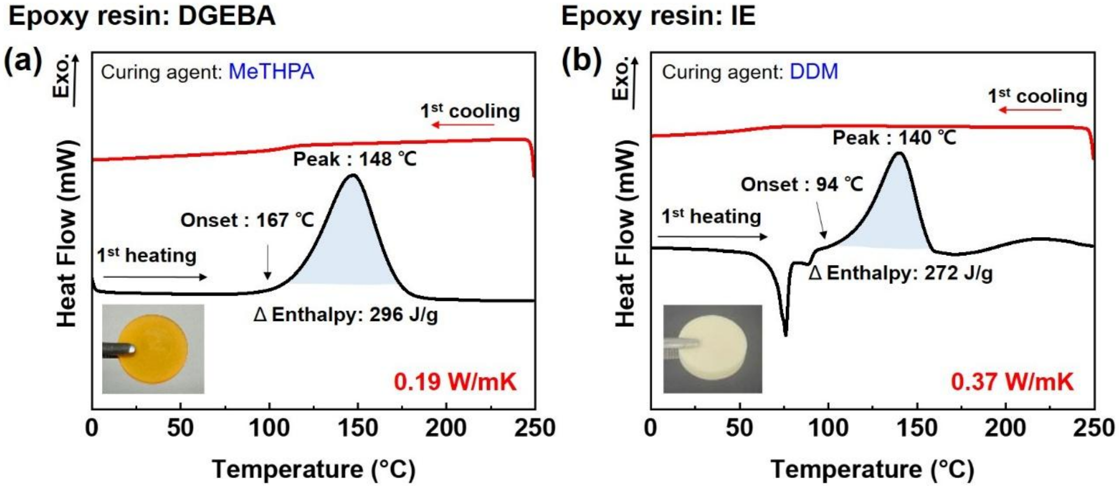

Figure S3. DSC first heating and cooling curves of (a) DGEBA/MeTHPA and (b) IE/DDM systems measured under nitrogen atmosphere at a heating rate of 10 °C min^{-1}. The thermal conductivity values determined for (a) and (b) were 0.1940 and 0.3700 W/m·K, respectively.

3.9 FT-IR Analysis of Vitrimer Reprocessing Cycles

The first- and fourth-generation vitrimer samples were analyzed by FT-IR spectroscopy to verify the preservation of dynamic imine bonds after repeated reprocessing. As shown in **Figure S4**, the characteristic C=N stretching vibration at ~1680 cm^{-1} remains clearly

observable with negligible change in intensity and position, indicating that the imine-linked network structure is chemically preserved during hot-press reprocessing. This confirms that the vitrimer network undergoes topological rearrangement via dynamic imine exchange rather than irreversible bond degradation.

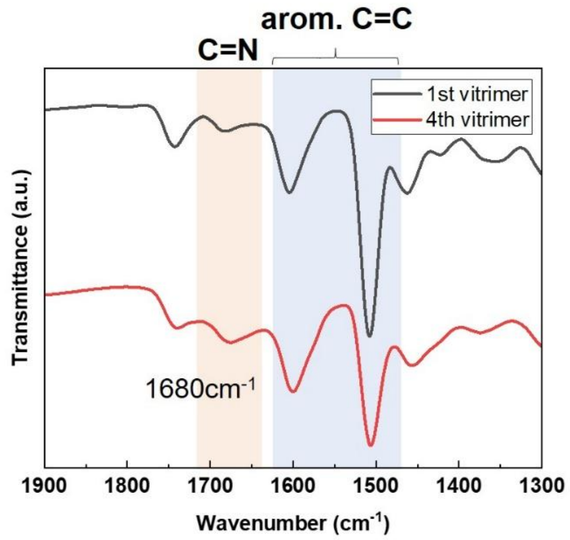

Figure S4. FT-IR spectra of the first- and fourth-generation vitrimer samples after repeated reprocessing cycles. The characteristic C=N stretching vibration (~1680 cm^{-1}) is clearly preserved with negligible change in both intensity and position, confirming that the imine-linked network structure is chemically maintained during hot-press reprocessing.

3.10 Procedure for curing IE composites with filler

To prepare filler-containing IE composotes, the imine-functionalized liquid crystalline epoxy (IE) resin was first thoroughly mixed with 3 parts per hundred resin (PHR) of N-TFSI, the cationic initiator. Subsequently, 3 PHR of 2-ethyl-4-methylimidazole (2E4MZ) catalyst was added and the mixture was further blended to ensure homogeneity. Finally, the filler was incorporated and thoroughly dispersed to obtain a uniform composite mixture.

Differential scanning calorimetry (DSC) was conducted to determine the approproate curing temperature. The first heating curve **(Figure S5)** showed an onset temperature of 125 °C and a peak temperatyre of 140 °C for the IE/N-TFSI/2E4MZ system. With the use of the catalyst, the curing region overlapped with the liquid crystal (LC) transition range, allowing for isothermal curing at 125 °C without the need for a separate two-step procedure.

The curing procedure that described in Section 3.7. The homogeneous mixture was transferred into a hot-press mold and cured under 5Mpa pressure for 3 h. Upon completion, the samples were cooled to room temperature, demolded, and polished to obtain the final filler-containing IE composite specimes.

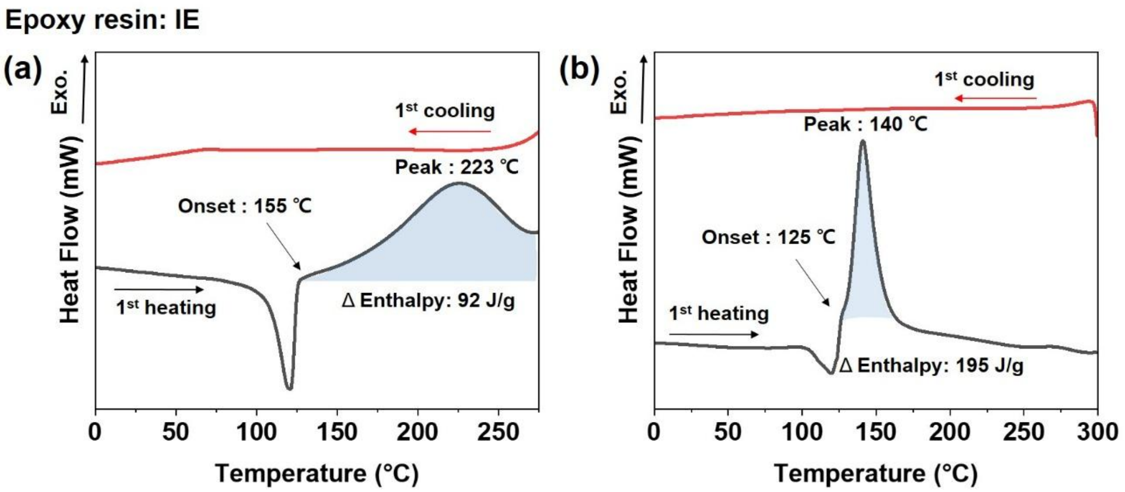

Figure S5. DSC first heating and cooling curves of (a) IE/N-TFSI (3 PHR) and (b) IE/N-TFSI (3 PHR)/2E4MZ (3 PHR) systems measured under nitrogen atmosphere at a heating rate of 10 °C·min^{-1}, showing the effect of the catalyst in curing behavior.

3.11 Comparison of theoretical models for thermal conductivity prediction

The effective thermal conductivity (*Kc*) of filler-reinforced epoxy composites was predicted using two representative theoretical models: the Lewis–Nielsen model and the Agari–Uno model. The Lewis–Nielsen model explicitly considers filler geometry through the aspect ratio, making it suitable for anisotropic fillers such as platelet-shaped *h*-BN, whereas the Agari–Uno model does not include a shape factor and is often applied as a semi-empirical model for various composite systems.

(1) Lewis-Nielsen model
The Lewis–Nielsen model is expressed as:

$$\frac{K}{K_m} = \frac{1+AB\Phi}{1-\ \psi B\Phi}$$

where K_m and K_f are the thermal conductivities of the matrix and filler, respectively, Φ is the filler volume fraction, Φ_m is the maximum packing fraction, A is the particle shape/orientation factor, B is the conductivity ratio factor, and ψ is the packing correction term.

The factors B and ψ are defined as:

$$B = \frac{\frac{Kf}{K_m} - 1}{\frac{Kf}{K_m} + A} \qquad \psi = 1 + \frac{1-\Phi m}{\Phi m2} \cdot \Phi$$

The geometry factor A is related to the aspect ratio P of the filler as follows:

$$A = PE - 1, \quad PE = \frac{P}{2\,ln(2P) - 3} + 2$$

(2) Agari-Uno model
The Agari–Uno model is a semi-empirical model that describes the thermal conductivity of composite systems without explicitly considering filler geometry.

The model is expressed as:

$$\log_{10} Kc = (1-\Phi)\log_{10}(C_1 K_m) + \Phi C_2 \log_{10} K_f$$

where K_c, K_m , and K_f are the thermal conductivities of the composite, matrix, and filler, respectively, Φ is the filler volume fraction, and C_1 and C_2 are empirical parameters representing the influence of the filler on the polymer structure and the formation of thermally conductive pathways, respectively.

The parameters C_1 and C_2 were determined by fitting the experimental thermal conductivity data to the Agari–Uno equation using a nonlinear least-squares optimization procedure, where C_1 and C_2 were treated as free fitting parameters. The filler volume fractions used in the fitting were converted from the weight fractions based on the densities of the matrix and the filler. Only the data points below 85 wt% filler loading were included in the fitting to exclude the strongly aggregated regime at extremely high filler contents, in which the model assumptions are no longer valid.

(3) Model comparison for *h*-BN-filled composites
For the Lewis–Nielsen calculations of the IE/*h*-BN composites, the thermal conductivity of the epoxy matrix (K_m) was taken as 0.86 W m^{-1} K^{-1}, and that of *h*-BN (K_f) was set to 400 W m^{-1} K^{-1} based on literature values. The maximum packing fraction Φ_m was fixed at 0.8, and aspect ratios P = 30 and 50 were used to represent the platelet geometry of *h*-BN[3].

For the Agari–Uno model, the same values of K_m and K_f were used as described above. The experimental thermal conductivity data below 85 wt% filler loading were fitted to the Agari–Uno equation to avoid the strongly aggregated regime at extremely high filler contents. As a result, the optimized fitting parameters were obtained as C_1=11.7 and C_2 =0.52.

Figure S6a–c compare the experimental thermal conductivity values of the IE/*h*-BN composites with the predictions obtained from the Lewis–Nielsen model using aspect ratios of P = 30 and 50, and from the Agari–Uno model, respectively. When aspect ratios of P = 30 and 50 are applied **(Figure S6a,b),** both Lewis–Nielsen predictions reproduce the overall increasing trend of the experimental data with comparable accuracy over the investigated filler content range. The model with P = 50 shows slightly better agreement, particularly at high filler loadings, and was therefore adopted as a representative effective aspect ratio for the present composites.

In contrast, the Agari–Uno model **(Figure S6c)**, which does not explicitly consider filler geometry, exhibits larger deviations from the experimental data, especially at high filler contents. These results indicate that explicit consideration of filler aspect ratio is necessary to properly describe the thermal transport behavior of platelet-shaped *h*-BN fillers, while the precise value of the effective aspect ratio (P = 30 or 50) has only a minor influence within this range.

It should also be noted that the experimental data point at 90 wt% *h*-BN shows an anomalously low thermal conductivity compared to the overall trend. This deviation is not an artifact of measurement but originates from severe processing and structural integrity issues at such an excessively high filler loading. As shown in **Figure S7**, the composite containing 90 wt% *h*-BN becomes extremely brittle and partially collapses during demolding and handling, indicating insufficient matrix continuity and poor structural integrity. This macroscopic failure directly explains the sharp drop in thermal conductivity at 90 wt%, as the excessive filler content disrupts the formation of continuous and mechanically stable thermal transport pathways.

(4) Model comparison for Al_2O_3-filled composites

For the Lewis–Nielsen calculations of the IE/Al_2O_3 composites, the thermal conductivity of the epoxy matrix (K_m) was taken as 0.86 W m^{-1} K^{-1}, and that of Al_2O_3 (K_f) was set to 30 W m^{-1} K^{-1}, which is a typical value for ~20 μm alumina particles. The maximum packing fraction Φ_m was fixed at 0.8 to represent the dense packing condition in the highly filled composite system. An aspect ratio P=1.2 was used to represent the nearly equiaxed particulate morphology of the alumina filler. This slightly larger-than-unity aspect ratio was adopted to account for the weak nonsphericity and irregular shape of the particles, which is a commonly used approximation for particulate ceramic fillers.

For the Agari–Uno model, the same values of K_m and K_f were used as described above. The experimental thermal conductivity data were fitted to the Agari–Uno equation using the filler volume fractions converted from the weight fractions based on a filler density of 3.95 g cm^{-3} and a matrix density of 1.20 g cm^{-3}. As a result, the optimized fitting parameters were obtained as C_1=2.61 and C_2=0.47.

To further validate the applicability of each model, the same analysis was performed for composites filled with spherical Al_2O_3 particles (average diameter ~20 μm). **Figures S6d** and **S6e** compare the experimental thermal conductivity values with the predictions obtained from the Lewis–Nielsen model using an aspect ratio of P = 1.2 and from the Agari–Uno model, respectively. In this case, the Lewis–Nielsen model shows noticeable deviation from the experimental data, whereas the Agari–Uno model provides a closer overall agreement across the investigated filler content range. This result is consistent with the nearly isotropic geometry of the spherical Al_2O_3 filler, for which explicit consideration of a high aspect ratio is unnecessary.

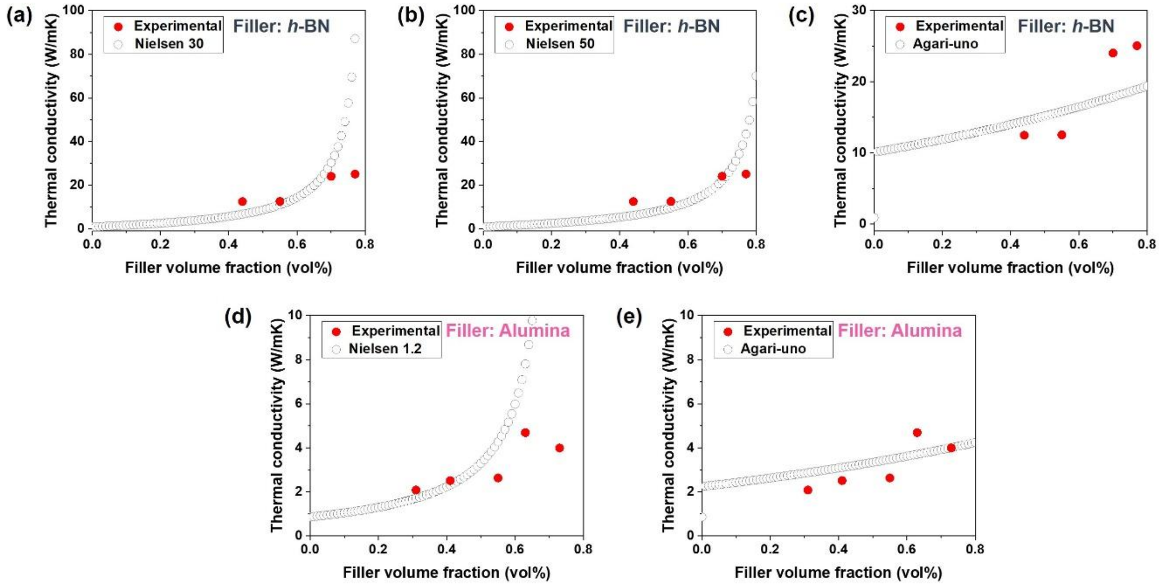

Figure S6. Comparison between experimental thermal conductivity values and model predictions for (a–c) IE/*h*-BN composites and (d,e) IE/Al_2O_3 composites. (a) Lewis–Nielsen model with P = 30 for *h*-BN-filled composites, (b) Lewis–Nielsen model with P = 50 for *h*-BN-filled composites, (c) Agari–Uno model for *h*-BN-filled composites, (d) Lewis–Nielsen model with P = 1.2 for Al_2O_3-filled composites, and (e) Agari–Uno model for Al_2O_3-filled composites.

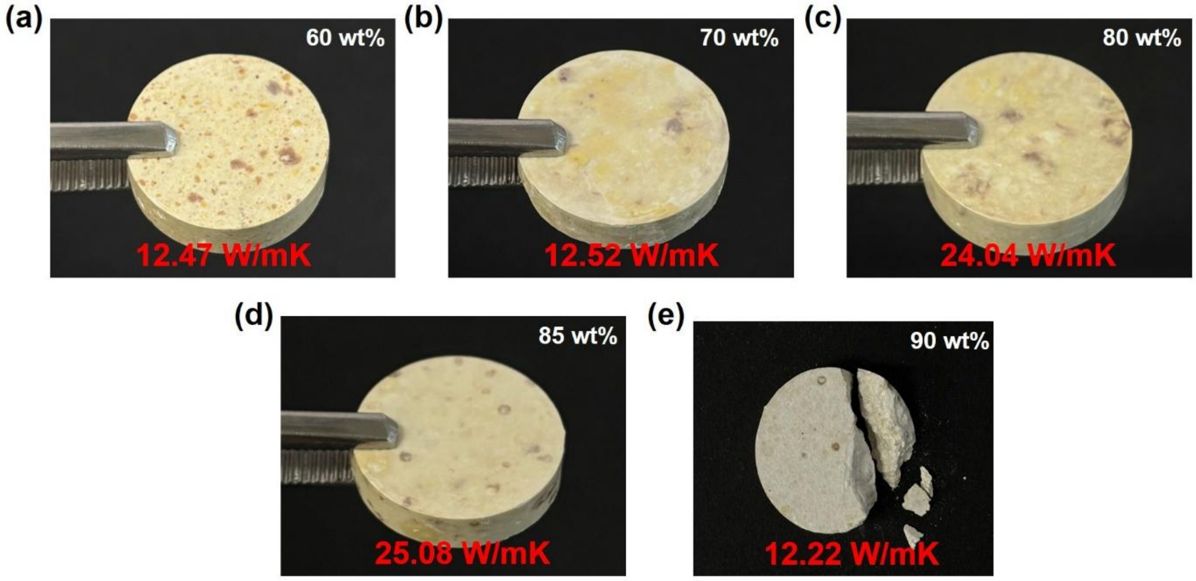

Figure S7. Photographs of the cured IE/*h*-BN composites containing (a) 60 wt%, (b) 70 wt%, (c) 80 wt%, (d) 85 wt%, and (e) 90 wt% *h*-BN filler. The corresponding thermal conductivity values are 12.47, 12.52, 24.04, 25.08, and 12.22 W m^{-1} K^{-1}, respectively. The composite with 90 wt% *h*-BN exhibits severe brittleness and partial structural collapse during demolding and handling, indicating insufficient matrix continuity at excessively high filler loading.

3.12 Kinetic analysis of curing behavior

3.12.1 Degree of conversion calculation

The curing progress of the imine-based liquid crystalline epoxy (IE) systems was evaluated by differential scanning calorimetry (DSC). The degree of conversion (α) was calculated from the DSC heat flow curves according to:

$$\alpha(T) = \frac{\Delta H_T}{\Delta H_{total}}$$

where ΔH_T is the cumulative reaction enthalpy up to temperature T, and ΔH_{total} is the total reaction enthalpy obtained by integrating the entire exothermic curing peak. No residual exothermic peak was observed in the second heating DSC scans, confirming that the curing reactions were essentially completed under the selected conditions.

Non-isothermal DSC measurements were performed at heating rates of 2.5, 5.0, 10, and 20 °C/min. The resulting conversion–temperature (α–T) curves for IE systems cured with N-TFSI and B-TFSI are shown in **Figure 6a–d**.

3.12.2 Isoconversional kinetic analysis using the KAS method

To quantitatively analyze the curing kinetics without assuming a specific reaction model, the Kissinger–Akahira–Sunose (KAS) isoconversional method was employed.

Under non-isothermal conditions with a constant heating rate $\beta = \frac{dT}{dt}$, the KAS method yields the following linearized equation for a fixed degree of conversion α:

$$\ln\left(\frac{\beta}{T\alpha^2}\right) = const - \frac{E_a}{RT_\alpha}$$

where T_α is the absolute temperature corresponding to a given conversion α, R is the gas constant, and E_a is the activation energy. For each conversion level (α = 10–90%), the values of $\ln(\beta/ T_\alpha{}^2)$ were plotted as a function of $1/T_\alpha$ using data obtained at four different heating rates.[4]

Representative KAS plots for the N-TFSI- and B-TFSI-cured systems are shown in **Figure 6e** and **Figure 6f**, respectively.

For both systems, overall linear trends are observed at each conversion level, confirming the applicability and validity of the KAS isoconversional analysis.

3.12.3 Activation energy profiles

The activation energy values were obtained from the slopes of the linear fits in the KAS plots according to:

$$E_a = -(slope)\ \times R$$

The resulting activation energy profiles as a function of degree of conversion are summarized in **Figure 6g**. Over the entire conversion range, the N-TFSI-cured system exhibits systematically higher activation energy values than the B-TFSI-cured system, indicating that the curing reaction proceeds with a higher energetic barrier when initiated by N-TFSI.

Notably, the activation energy of the N-TFSI system changes more gradually with increasing conversion, whereas the B-TFSI system shows relatively larger variation in activation energy as the reaction progresses. This difference suggests distinct kinetic behaviors governing the curing processes of the two initiator systems.

3.12.4 Isothermal DSC analysis at 220 °C

To further examine the intrinsic kinetic persistence of the initiator systems under conditions where diffusion-related and structural effects are minimized, isothermal DSC measurements were conducted at 220 °C. This temperature was intentionally selected not to represent the practical curing condition, but to provide a reference state that enables a clearer comparison of initiator-controlled reaction persistence. The corresponding heat flow curves are presented in **Figure 6h**.

As shown in **Figure 6h**, the N-TFSI-cured system exhibits a more gradual decay of heat flow and reaches reaction completion at a later time compared to the B-TFSI-cured system. This behavior directly demonstrates that the curing reaction initiated by N-TFSI proceeds in a more sustained manner, reflecting a longer effective initiator lifetime. Importantly, the sustained curing behavior observed at 220 °C reveals the intrinsic kinetic origin that underpins the curing behavior and structural development observed under practical curing conditions.

3.12.5 Overall interpretation

Taken together, the non-isothermal conversion behavior **(Figure 6a–d)**, the linear KAS plots **(Figure 6e,f)**, the activation energy profiles **(Figure 6g)**, and the isothermal DSC results **(Figure 6h)** consistently demonstrate that the N-TFSI initiator exhibits moderated reactivity and a prolonged kinetic lifetime compared to B-TFSI.

In particular, the high linearity of the KAS plots observed for the N-TFSI system indicates that the dominant kinetic mechanism remains relatively uniform over the entire conversion range, whereas the slight curvature observed in the B-TFSI system suggests a gradual change in the kinetic regime as the reaction proceeds. This difference can be attributed to the enhanced stabilization of the cationic active species by the naphthalene moiety in N-TFSI, which enables a more controlled and sustained curing process.

4. References

[1] X. Xu, S. Ma, J. Wu, J. Yang, B. Wang, S. Wang, Q. Li, J. Feng, S. You, J. Zhu, J. High-performance, command-degradable, antibacterial Schiff base epoxy thermosets: synthesis and properties, Mater. Chem. A **2019**, 7, 15420

[2] J. Kim, S. Kim, S. K. Choi, S. Yang, M. Kim, Regulating Cationic Polymerization of Difunctional Epoxy Resin through Structural Variations of the Thermal Initiator, ACS Appl. Polym. Mater. **2024**, 6, 6689

[3] A. M. Islam, H. Lim, N.-H. You, S. Ahn, M. Goh, J. R. Hahn, H. Yeo, S. G. Jang, Enhanced Thermal Conductivity of Liquid Crystalline Epoxy Resin using Controlled Linear Polymerization, ACS Macro Lett. **2018**, 7, 1180.

[4] G. Mashouf Roudsari, A. K. Mohanty, M. Misra, Study of the Curing Kinetics of Epoxy Resins with Biobased Hardener and Epoxidized Soybean Oil, ACS Sustain. Chem. Eng. **2014**, 2, 2111–2116.
